# Dysregulated mitochondrial homeostasis and DNA repair in the progression from colon adenoma to cancer

**DOI:** 10.1186/s10020-025-01400-5

**Published:** 2025-11-22

**Authors:** Natalie Danesova, Josef Horak, Anna Valickova, Adrian Gil-Korilis, Jorge Ergui-Arbizu, Richard Palek, Jan Bruha, Miroslav Levy, Pavel Skrobanek, Jan Kral, Jiri Jungwirth, Jiri Neuzil, Veronika Vymetalkova, Pavel Vodicka, Sona Vodenkova, Kristyna Tomasova

**Affiliations:** 1 Department of Molecular Biology of Cancer, Institute of Experimental Medicine of the Czech Academy of Sciences, Prague, Czech Republic; 2 Biomedical Centre, Faculty of Medicine in Pilsen, Charles University, Pilsen, Czech Republic; 3 Molecular Targets Program, Center for Cancer Research (CCR), National Cancer Institute (NCI), National Institutes of Health (NIH), Frederick, MD USA; 4 Institute of Biology and Medical Genetics, First Faculty of Medicine, Charles University, Prague, Czech Republic; 5 Department of Surgery, Medical Faculty in Pilsen, Charles University, Pilsen, Czech Republic; 6 Department of Surgery, First Faculty of Medicine, Charles University and Thomayer Hospital, Prague, Czech Republic; 7 Department of Oncology, First Faculty of Medicine, Charles University and Thomayer Hospital, Prague, Czech Republic; 8 Department of Internal Medicine, University Hospital Motol, Second Faculty of Medicine, Charles University, Prague, Czech Republic; 9 Department of Hepatogastroenterology, Institute for Clinical and Experimental Medicine, Prague, Czech Republic; 10 Institute of Physiology, First Faculty of Medicine, Charles University, Prague, Czech Republic; 11 Department of Gastroenterology, Libera Scientia, Prague, Czech Republic; 12 Laboratory of Molecular Therapy, Institute of Biotechnology, Czech Academy of Sciences, Prague-West, Czech Republic; 13 Department of Genetics and Microbiology & Department of Physiology, Faculty of Science, Charles University, Prague, Czech Republic; 14 Department of Paediatrics and Inherited Metabolic Disorders, First Faculty of Medicine, Charles University, Prague, Czech Republic; 15 School of Pharmacy and Medical Science, Griffith University, Southport, Qld Australia

**Keywords:** Colorectal cancer, Colon adenomas, Biomarkers, Mitochondria, Mitochondrial DNA damage, Mitochondrial DNA repair, Mitochondrial DNA copy number

## Abstract

**Background:**

While nuclear DNA (nDNA) damage and alterations in nDNA repair are known to play a role in colon cancer (CC), there is insufficient research investigating these processes in mitochondrial DNA (mtDNA).

**Methods:**

This study investigates mtDNA changes in CC, focusing on mitochondrial DNA copy number (mtDNA-CN) variations, mtDNA damage, and the expression and mutation status of DNA repair genes. Three cohorts were analyzed: healthy controls, colon adenoma patients, and CC patients, divided into a pilot and a validation set.

**Results:**

Our findings revealed that mtDNA-CN was elevated in colon adenomas compared to adenoma-adjacent mucosa (FDR = 0.04), healthy mucosa (FDR = 0.005), and tumor-adjacent mucosa (FDR = 0.005). Moreover, mtDNA-CN was elevated in adenoma-adjacent mucosa compared to healthy mucosa (FDR = 0.04). MtDNA damage was greater in tumor-adjacent mucosa compared to tumor tissue in both the pilot and validation sets (FDR = 0.031 and FDR = 2.06e-05, respectively). Additionally, we identified novel DNA repair genes associated with mtDNA damage, predominantly upregulated in adenoma and tumor tissues compared to healthy colon tissues.

**Conclusions:**

To conclude, this study highlights the importance of mtDNA alterations in CC development and identifies potential mtDNA biomarkers.

**Supplementary Information:**

The online version contains supplementary material available at 10.1186/s10020-025-01400-5.

## Introduction

Mitochondria, while being the powerhouses of the cell and regulators of redox homeostasis, play a pivotal role in tumor development through oncogenic signaling, metabolic reprogramming, oxidative stress regulation, and apoptotic resistance (Annesley and Fisher 2019; Tait and Green 2012). Mitochondria possess their own DNA, i.e. mitochondrial DNA (mtDNA), which encodes key components of the respiratory chain (Sharma et al. 2019). Unlike diploid nuclear DNA (nDNA), mtDNA is present in multiple copies per cell, is more susceptible to damage, and its repair mechanisms are not well understood (Vodicka et al. 2024).

In cancer, mtDNA alterations are frequent, including changes in the total number of mtDNA copies per cell, referred to as mtDNA copy number (mtDNA-CN) or mtDNA content (Filograna et al. 2021). Changes in mtDNA-CN have been reported in colorectal cancer (CRC), one of the most frequent cancers worldwide (Bray et al. 2024), with studies showing both increased and decreased levels (Osch et al. 2015; Qu et al. 2015; Wang et al. 2016). Much of the existing research has focused on peripheral blood, suggesting that leukocyte mtDNA-CN could serve as a biomarker for CRC risk or early detection (Yang et al. 2019; Thyagarajan et al. 2016; Huang et al. 2014). Among the CRC tissue-based studies, one found that low mtDNA-CN in tumors was linked to worse prognosis in CRC (Osch et al. 2015). Another study reported that mtDNA-CN levels correlated with the prognosis of CRC stage II and III patients with deficient mismatch repair (MMR) (Chen et al. 2024). Moreover, there are indications that mitochondrial transcription factor A (TFAM) and p53 expression may increase mtDNA-CN in tumors (Sun et al. 2018). Overexpression of *TFAM* has been observed in microsatellite-stable (MSS) CRC and resulted in increased mtDNA-CN (Sun et al. 2018). An increase in mtDNA-CN is thought to compensate for mtDNA damage or mitochondrial dysfunction caused by impaired respiratory function or mutations (Abd Radzak et al. 2022).

mtDNA is highly vulnerable to damage due to the absence of protective histones, its proximity to reactive oxygen species (ROS), a higher mutation rate during replication, and limited DNA repair capacity (Lax et al. 2011). Unlike the nucleus, mitochondria lack the nucleotide excision repair (NER), making certain types of mtDNA damage irreparable (Liao et al. 2022). The predominant mtDNA repair pathway is base excision repair (BER), removing lesions caused by oxidation, alkylation, methylation, and deamination (Stein and Sia 2017). Additionally, there is evidence suggesting the involvement of other repair pathways in mitochondria, such as MMR, double-strand break (DSB) repair, and direct reversal (DR) repair (Liao et al. 2022).

The most prevalent form of mtDNA damage is oxidative damage, primarily resulting from increased ROS production during ATP synthesis (Kowalska et al. 2020). Other recognized forms of mtDNA damage are alkylation, DNA adduct formation, and single-strand breaks (SSBs) or DSBs (Bont and Larebeke 2004; Blair 2008). One study reported more prominent mtDNA damage in rectal cancer compared to colon malignancy (Potenza et al. 2011). Frequent hotspot mtDNA mutations in CRC include alterations in the NADH dehydrogenase subunit 1 (MT-ND1) (Brandt 2006), potentially leading to impaired ATP synthesis, ROS overproduction, and increased oxidative stress (Beckman and Ames 1997). Elevated levels of circulating *MT-ND1* have been linked to poor prognosis in CRC (Xu et al. 2021). Another common mutation in CRC, the 4977-bp mtDNA deletion, also disrupts ATP synthesis (Beckman and Ames 1997; Xu et al. 2021; Dani et al. 2003), contributing to errors during mtDNA replication and DSB repair (Chen et al. 2011). However, broader patterns of mtDNA damage and its repair in CRC remain largely unexplored.

In this study, we explored the potential colon cancer (CC) biomarkers associated with mtDNA, focusing on mtDNA-CN, mtDNA damage, and relevant repair processes. Although mitochondrial dysfunction and alterations in mtDNA-CN have been linked to CRC in several studies, much less is known about their role in premalignant adenomas and throughout the adenoma–carcinoma sequence. We hypothesized that changes in mtDNA-CN, mtDNA damage and associated DNA repair expression occur already at the colon adenoma stage and may contribute to the early steps of tumorigenesis.

By systematically analyzing all mtDNA-CN, mtDNA damage and markers of DNA repair in adenomas, adjacent mucosa, and tumors, our study provides novel insights into mitochondrial alterations in precancerous colon lesions, highlighting their potential as biomarkers for the early detection and risk stratification of CC. Unlike previous research primarily focused on isolated mtDNA mutations or alterations in peripheral blood, our study provides a comprehensive assessment of mtDNA damage, including mtDNA strand-breaks and deletions. To the best of our knowledge, this is the first study to apply such an approach in both colon adenomas and CC, offering new insights into mitochondrial biology and colon tumorigenesis. By integrating analyses of mtDNA-CN, mtDNA damage, and repair processes across the entire progression from healthy tissue through colon adenoma to CC, our study contributes to a better understanding of mitochondrial involvement in CC pathogenesis and its potential for biomarker development.

## Methods

### Sample collection and processing

**Fig. 1:**
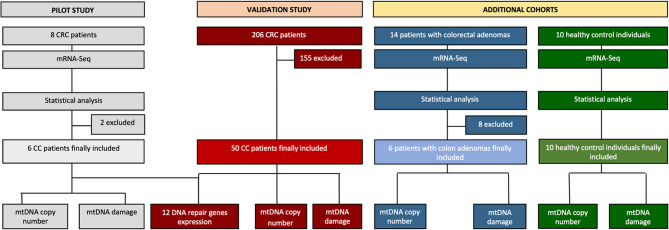
The study workflow. The study consisted of two main phases involving CC patients: a pilot study followed by a validation study. To enable a comparison, two additional cohorts were included: control individuals and patients with precancerous lesions (adenomas). Both CC and colon adenoma patients were included based on specific inclusion criteria, including no prior history of cancer or oncological treatment, defined disease type and location, and sufficient nucleic acid quality. The healthy controls were individuals with no personal history of cancer. Abbreviations: CC – colon cancer, mtDNA – mitochondrial DNA, mRNA-Seq – messenger RNA sequencing

A total of 56 CC patients were included in this study, which was conducted in two phases: a pilot study and a validation study, as outlined in the Study Workflow (Fig. 1).

- *Pilot study*: Six newly diagnosed, histologically confirmed CC patients with no prior cancer, oncological treatment, or CRC attributed to well-defined hereditary syndromes were enrolled between 2022 and 2023 at the Department of Surgery, University Hospital in Pilsen, Czech Republic. Paired tumor and adjacent non-malignant mucosa samples were snap-frozen post-surgery in liquid nitrogen and stored at −80 °C.
- *Validation Study:* To validate the pilot findings, fifty CC patients treated between 2010-2020 at the Department of Surgery, Thomayer University Hospital in Prague, Czech Republic, were selected using the same inclusion criteria. Rectal cancer patients were excluded due to different treatment strategies, which could confine the results. The samples were processed identically to the pilot set.
- *Additional Cohorts:* To examine mtDNA changes in healthy and precancerous colon tissues, two additional cohorts were included. The first cohort comprised ten healthy control individuals with no personal history of cancer and no polyps or neoplastic lesions found during colonoscopy screening; these individuals provided healthy colon mucosa samples. The second cohort consisted of six patients with colon adenomas, also with no personal history of cancer or oncological treatment, with defined disease type and location; these patients provided paired adenoma and adjacent non-affected mucosa samples. Both cohorts were recruited between 2020-2023 at the Clinic of Hepatogastroenterology, Institute for Clinical and Experimental Medicine in Prague, Czech Republic.

Fresh-frozen tissue material was surgically collected at three hospitals following standard procedures. After macroscopic inspection, ~ 3 × 3 mm pieces of viable tumor tissue and adjacent non-malignant mucosa (5–15 cm from the tumor margin) were excised, placed into cryovials, and immediately frozen at −80 °C until nucleic acid extraction. Adenoma tissue, their adjacent non-affected mucosa (5 cm from the lesion), and colon mucosa from healthy control individuals were collected using the same approach. Sociodemographic and clinicopathological data were obtained from structured questionnaires and/or medical records. Participants signed a written informed consent to participate in the study and agreed to the use of their biological samples according to the Helsinki Declaration. The study was approved by the Ethics Committee of the Institute for Clinical and Experimental Medicine and Thomayer University Hospital, Prague, Czech Republic (6799/21 G-20–55, 06366/23 + 15408/23; A-23-07, protocol N. G-17-03-02), and the Ethics Committee of the Pilsen University Hospital, Pilsen, Czech Republic (120/2021, 250/2021). The characteristics of CC patients (pilot and validation sets), colon adenoma patients, and healthy controls are presented in Table 1.

**Table 1.** Characteristics of CC patients, adenoma patients, and control individuals included in the study

CATEGORY	PARAMETER	CC PATIENTS PILOT SET *n* (%)	CC PATIENTS VALIDATION SET *n* (%)	ADENOMA PATIENTS *n* (%)	CONTROL INDIVIDUALS *n* (%)
Total number of individuals		6	50	6	10
Sex					
	Males	3 (50.0)	30 (60.0)	4 (66.7)	6 (60.0)
	Females	3 (50.0)	20 (40.0)	2 (33.3)	4 (40.0)
Age at the time of diagnosis (years)		Median [range] 74 [41-87]	Median [range] 71 [40-85]	Median [range] 60.5 [50-69]	Median [range] 55 [40-61]
	40-60		8 (16.0)	3 (50.0)	8 (80.0)
	60-80		33 (66.0)	3 (50.0)	2 (20.0)
	80-100		9 (18.0)		
BMI (kg/m^2^)					
	<25	N/A	11 (22.0)		3 (30.0)
	25-30	N/A	20 (40.0)	3 (50.0)	5 (50.0)
	>30	N/A	19 (38.0)	3 (50.0)	2 (20.0)
Tumor location^1^					
	Proximal colon (C18-18.5)	5 (83.3)	20 (40.0)		
	Distal colon (C18.6-C19)	1 (16.7)	30 (60.0)		
	Rectum (C20)	0	0		
TNM stage^2^					
	1	0	6 (12.0)		
	2	2 (33.3)	22 (44.0)		
	3	4 (66.7)	16 (32.0)		
	4	0	6 (12.0)		
Tumor grade					
	1	3 (50.0)	0		
	2	2 (33.3)	23 (46.0)		
	2-3	0	10 (20.0)		
	3	0	17 (34.0)		
	Not determined	1 (16.7)	0		
MSI status					
	Stable	N/A	25 (50.0)		
	Low	N/A	2 (4.0)		
	High	N/A	3 (6.0)		
	Undetected	N/A	20 (40.0)		
CC therapy					
	No Therapy	5 (83.3)	22 (44.0)		
	Adjuvant Therapy	1 (16.7)	28 (56.0)		
CC recurrence					
	No	N/A	47 (94.0)		
	Yes	N/A	3 (6.0)		
Type of adenoma					
	Tubular			4 (66.7)	
	Tubolovillous			2 (33.3)	
Adenoma location					
	Distal colon			6 (100)	
Adenoma grade					
	Low-grade dysplasia			5 (83.3)	
	High-grade dysplasia			1 (16.7)	

CC patients were divided into two distinct groups: a pilot and a validation set

*Abbreviations*: *BMI* body mass index, *CC* colon cancer, *MSI* microsatellite instability, *N/A* not available, *TNM* tumor-node-metastasis

^1^Tumor location was differentiated based on the International Statistical Classification of Diseases and Related Health Problems, 10th revision (ICD-10) codes (WHO, 2004)

^2^TNM (tumor-node-metastasis) classification was used for colon cancer staging

### DNA and RNA extraction

DNA and RNA were extracted from all tissue samples (healthy mucosa, paired adenoma and adjacent non-affected mucosa, paired tumor and adjacent non-malignant mucosa) by the QIAamp DNA Mini Kit and RNeasy Mini Kit (both Qiagen, Germany) using the protocol provided by the manufacturer. DNA and RNA concentrations were assessed using the NanoDrop 2000 spectrophotometer (Thermo Fisher Scientific, USA). DNA samples were stored at −20 °C, RNA samples at −80 °C.

### Measurement of mitochondrial DNA copy number

Relative mtDNA-CN was quantified by multiplex quantitative polymerase chain reaction (qPCR) in 384-well plates using Taqman probes (Thermo Fisher Scientific, USA). The mitochondrial gene NADH dehydrogenase 1 (*MT-ND1*, Hs02596873_s1, VIC) expression was quantified relative to the nuclear housekeeping genes beta-2-microglobulin (*B2M*, Hs00112422_cn, FAM) and ribonuclease P RNA component H1 (*RPPH1*, catalog number 4403326, VIC dye-labeled TAMRA). Reactions were performed on a QuantStudio^TM^6 Flex Real-Time PCR System (Thermo Fisher Scientific, USA) with the following cycling profile: 50 °C for 2 min, 95 °C for 10 min, and 40 cycles of 95 °C for 15 s and 60 °C for 1 min. The 10 ml reaction mixture included 5 ml of Taqman Universal Master mix II, no UNG (Applied Biosystems, USA), 3 ml of RNAse-free water, 0.5 ml of each probe, and 1 ml of 5 ng/ml DNA. Reported values represent relative expression in the form of 2^-ΔCt and are shown as median (interquartile range).

### Measurement of mitochondrial DNA damage

mtDNA damage was analyzed using the Human Real-Time PCR Mitochondrial DNA Damage Analysis Kit (Detroit R&D, USA), targeting an 8.8 kb mtDNA sequence. The analysis involved the preamplification of 8.8 kb mtDNA by PCR in a MiniAmp thermal cycle (Thermo Fisher Scientific, USA), followed by qPCR quantification on a QuantStudio^TM^6 Flex Real-Time PCR System (Thermo Fisher Scientific, USA). The PCR reaction (20 ml) included 10 ml of 2X qPCR buffer with polymerase, 4 ml of 5X enhancer, 5 ml of qPCR primer mix, and 1 ml of 50 ng/ml DNA, with cycling conditions of 98 °C for 30 s, 30 cycles of 98 °C for 10 s, 68 °C for 10 s, and 72 °C for 4 min, finishing at 72 °C for 10 min. The following qPCR reaction (20 ml) comprised of 10 ml of 2X Maxima SYBR green/Rox qPCR Master Mix (Thermo Fisher Scientific, USA), 8.1 ml of RNAse-free water, 0.9 ml of Real-Time primer mix, and 1 ml of ten times diluted PCR DNA product, with cycling at 50 °C for 2 min, 95 °C for 10 min, followed by 40 cycles of 95 °C for 15 s and 60 °C for 1 min. Each plate included control samples for the calibration curve provided with the kit. These control samples were used as interplate controls and to adjust the Ct values. Reported values represent relative expression in the form of 2^adjusted Ct value and are presented as median (interquartile range).

### mRNA sequencing

The RNA integrity number (RIN) of six CC samples from the pilot set (mean RIN = 7.14, see Additional Table 1A), six colon adenoma samples (mean RIN = 8.88, see Additional Table 1B), and ten healthy colon samples (mean RIN = 9.22, see Additional Table 1 C) was assessed using the Agilent RNA 6000 Nano Kit Quick Start Guide (Agilent, USA) following the manufacturer’s instructions, and measured on a Bioanalyzer (Agilent, USA). Libraries were prepared using the NEBNext mRNA Magnetic Isolation Module (New England BioLabs, USA) and proceeded to the second phase of library preparation using the NEBNext Ultra II Directional RNA Library Prep Kit for Illumina (New England Biolabs, USA), with ligation using NEBNext Multiplex Oligos for Illumina (Unique Dual Index Primer Pairs, New England Biolabs, USA). Sequencing was performed on a NovaSeq6000 S1 flow cell (Illumina, USA) with PE150, generating 2,600-3,200 million reads. All samples were pooled and resulted in 39–48 million reads.

### Transcriptome analysis using mRNA sequencing

Raw RNA sequencing data were processed with FastQC v0.11.9 and MultiQC v1.21 for quality control, Trimmomatic v0.39 for data trimming, BBmap v31.32 for ribosomal RNA filtering, STAR 2.7.10a for alignment, and RSEM v1.3.3 for quantification. Multidimensional scaling (MDS) confirmed the expected biological differences between samples over any technical issues (Additional Fig. 1). Differential gene expression was identified using EdgeR. Genes with an adjusted false discovery rate (FDR) < 0.05 (Benjamini-Hochberg procedure) were considered deregulated. Gene expression calculations were performed in RStudio 2023.6.1:524 (RStudio, USA).

### Validation of candidate DNA repair genes from mRNA sequencing by reverse transcription qPCR

The expression of twelve candidate DNA repair genes identified by messenger RNA Sequencing (mRNA-Seq) and associated with mtDNA damage in the pilot set was validated by reverse transcription qPCR (RT)-qPCR in 50 paired tumor and adjacent non-malignant tissue samples. RNA was reverse-transcribed into complementary DNA (cDNA) using the High-Capacity cDNA Reverse Transcription Kit with RNase Inhibitor (Thermo Fisher Scientific, USA) following the manufacturer’s protocol. Reverse transcription was conducted on a MiniAmp Thermal Cycler (Thermo Fisher Scientific, USA) with the following temperature profile: 25 °C for 10 min, 37 °C for 120 min, 85 °C for 5 min, then held at 4 °C. Reactions ran in a 20 ml mixture containing 10 ml of 90 ng/ml RNA, 2 ml of 10X RT Buffer, 0.8 ml of 100 mM 25X dNTPs, 2 ml of 10X Random Primers, 3.2 ml of RNAse-free water, 1 ml of RNAse Inhibitor, and 1 ml of MultiScribe Reverse Transcriptase (50 U/ml). cDNA was stored at −20 °C.

Gene expression was quantified in triplicates by the TaqMan Real-Time PCR Assays (Thermo Fisher Scientific, USA, Assays list provided in Additional Table 2) in 384-well plates on the QuantStudio™ 6 Flex Real-Time System (Thermo Fisher Scientific, USA). The qPCR conditions were: 50 °C for 2 min, 95 °C for 10 min, and 40 cycles of 95 °C for 15 s and 60 °C for 1 min. Each 10 ml qPCR reaction contained 5 ml of TaqMan Universal Master Mix II, no UNG (Thermo Fisher Scientific, USA), 3.5 ml of RNAse-free water, 0.5 ml of specific probe (Thermo Fisher Scientific, USA), and 1 ml of 20 ng/ml cDNA.

### mRNA sequencing-based detection of DNA mutations

DNA mutations were identified from the mRNA sequencing data. The raw data processing up to the alignment step is described in Sect. 2.6. Variant calling was performed using the GATK software and pipeline for somatic short variant discovery (Auwera and O’Connor 2020). Raw variants were filtered based on (i) a minimum site coverage of 30 reads and (ii) a variant allele frequency above 10%. Described artifacts, common population variants, and germline mutations were excluded using an automated filtering process. For comparative analyses, only mutations with a moderate or high predicted impact (missense, nonsense, frameshift) were considered. Synonymous mutations and those in regulatory regions (3´ UTR) were excluded.

### Statistical analysis

Statistical analyses were conducted using the R programming language (R version 4.3.1) and RStudio (version 2023.6.1:524, RStudio, USA). Normality of the data was assessed by the Shapiro–Wilk test. Multiple tests were performed to obtain the presented results, including the Wilcoxon signed-rank test for two-group comparisons and Spearman’s rank-order correlation coefficient or Kendall correlation test for summarizing the strength and direction (negative or positive) of the association between two ranked variables. DE analysis was then used to identify significantly deregulated genes between tumors and adjacent mucosa. Overall survival (OS) was estimated using multivariable Cox proportional-hazards models. Gene expression comparisons were conducted using a likelihood ratio test in EdgeR. The Benjamini-Hochberg procedure was used to control false discovery rate (FDR) and to avoid errors in multiple group comparisons.

Statistical significance thresholds were set at FDR < 0.05 (*), FDR < 0.01 (**), FDR < 0.001 (***), and FDR < 0.0001 (****).

## Results

### The mtDNA damage in tumor and adjacent mucosa and its association with the expression of DNA repair genes in the pilot and validation sets

In the pilot set of patients, mtDNA damage was significantly lower in tumors than in the adjacent mucosa [Wilcoxon test, 94.70 (813.95-125.74.95.74) and 1135.10 (766.84–3624.76.84.76), respectively, FDR = 0.031, Additional Fig. 2A]. This finding was confirmed in the validation set [Wilcoxon test, 107.63 (70.97–276.91.97.91) in tumors and 969.88 (285.75–4190.04.75.04) in adjacent mucosa, FDR = 2.06e-05, Additional Fig. 2B].

Based on these mtDNA damage results, we investigated whether mtDNA damage correlated with the expression of DNA repair genes, whose products are most likely also localized to mitochondria. We further identified DNA repair genes whose expressions not only correlated with mtDNA damage but also exhibited differential expression between the tumor and the adjacent mucosa. In the pilot study, the genes were categorized into functional pathways using the KEGG database. Only DNA repair pathways with a confirmed or likely mitochondrial role – BER, homologous recombination (HR), MMR, and non-homologous end joining (NHEJ) were considered, as described in (Vodicka et al. 2024).

Among the analyzed genes, 43 showed a correlation with mtDNA damage (Spearman´s rank-order correlation, all FDR ≤ 0.048, see Additional Table 3). Of these, 37 also exhibited different expression levels between the tumors and the adjacent mucosa (Spearman´s rank-order correlation, all FDR ≤ 0.021, see Additional Table 4). Of these 37 genes, 34 showed an inverse association between their expression and mtDNA damage, with higher expression in tumors compared to the adjacent mucosa. Conversely, three genes – *RAD52*, *NEIL1*, and *POLD4* – showed a positive correlation of their expressions with mtDNA damage and were more highly expressed in the adjacent mucosa.

Based on the pilot findings, 12 DNA repair genes with a moderate to strong correlation to mtDNA damage (correlation coefficient threshold of 0.6 (Akoglu 2018; Papageorgiou 2022), see Additional Table 3) were included in the validation analysis: *LIG3*,* RAD50*,* NEIL1*,* BRCA1*,* BRCA2*,* LIG1*,* MRE11*,* UNG*,* PARG*,* NEIL3*, and *POLD4*. Their involvement in specific DNA repair pathways is illustrated in Additional Fig. 3.

**Fig. 2:**
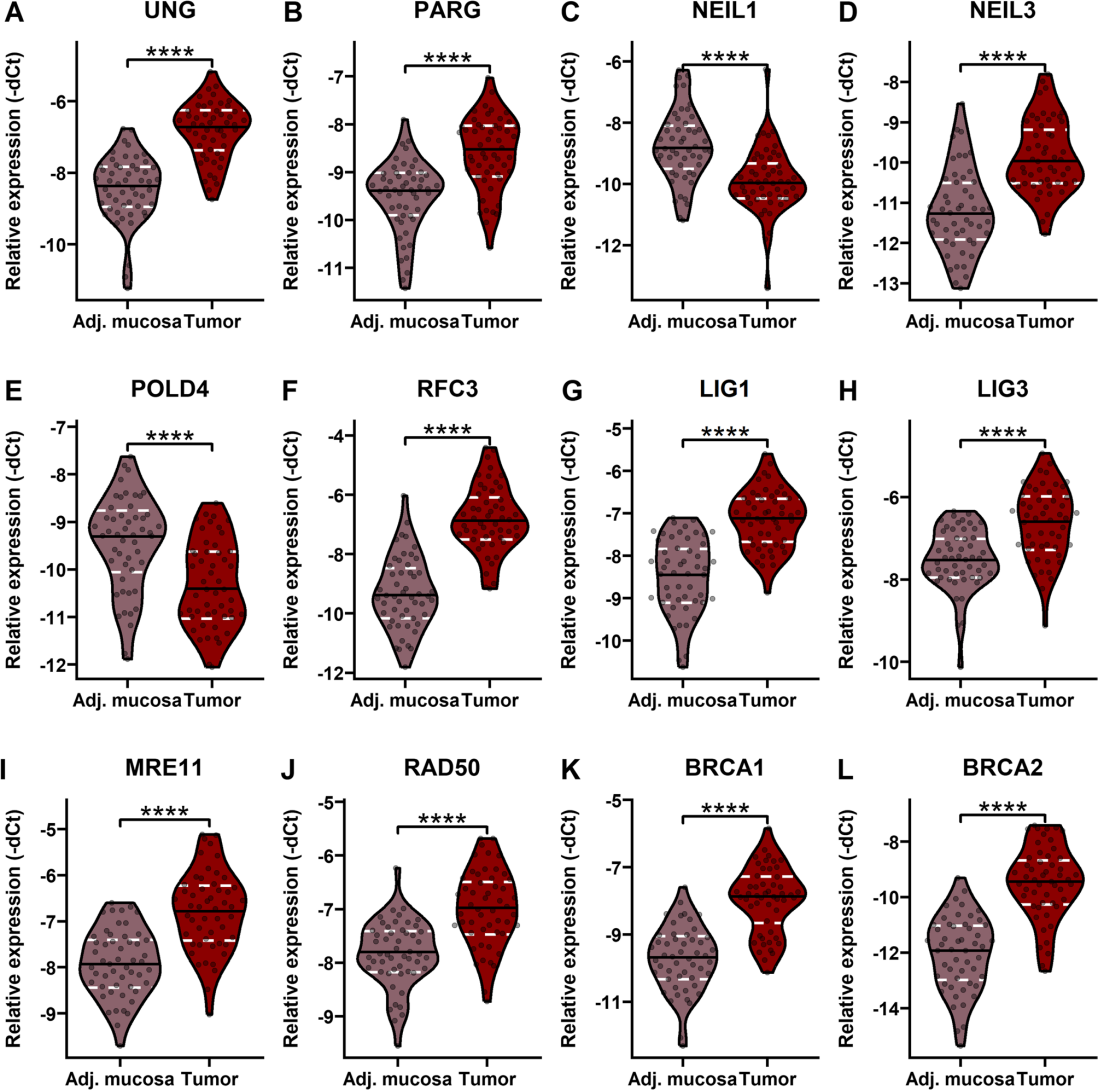
A comparison of the expression levels of 12 selected DNA repair genes (*UNG*, *PARG*, *NEIL1*, *NEIL3*, *POLD4*, *RFC3*, *LIG1*, *LIG3*, *MRE11*, *RAD50*, *BRCA1*, and *BRCA2*; panels A–L) between tumor and adjacent non-malignant mucosa in the validation set. The results confirmed the same trend of differential DNA repair gene expression levels between tumors and adjacent mucosa observed in the pilot study. **** FDR < 0.0001. Abbreviations: Adj. mucosa – adjacent mucosa

In the validation set, 11 of the 12 DNA repair genes (*BRCA1*, *BRCA2*, *LIG1*, *LIG3*, *MRE11*, *NEIL1*, *NEIL3*, *POLD4*, *RAD50*, *RFC3*, *UNG*), excluding *PARG*, remained significantly correlated with mtDNA damage (Spearman´s rank-order correlation, see Additional Table 5). All these 12 genes also exhibited significantly different expressions in tumors compared to the adjacent non-malignant mucosa (Wilcoxon test, see Additional Table 6), consistent with the pilot findings. Ten genes (*UNG*,* PARG*,* NEIL3*,* RFC3*,* LIG1*,* LIG3*,* MRE11*,* RAD50*,* BRCA1*, and *BRCA2*, see Fig. 2A, B, D, F-L) were upregulated in tumors (all FDR < 0.0001, see Additional Table 6), while two genes (*NEIL1* and *POLD4*, see Fig. 2C and E) were overexpressed in the adjacent mucosa (both FDR < 0.0001, see Additional Table 6). No associations were found between mtDNA damage and patient characteristics such as sex, age, body-mass index (BMI), tumor location, tumor-node-metastasis (TNM) stage, tumor grade, and OS (all FDR > 0.05).

### The mtDNA copy number in tumor and adjacent mucosa in the pilot and validation sets

In the pilot set, the mtDNA-CN levels showed no difference between tumors and adjacent mucosa [Wilcoxon test, 1518.00 (1252.90–2047.94.90.94) and 1102.61 (1073.98–1232.66.98.66), respectively, FDR > 0.05, Additional Fig. 4A], and did not correlate with mtDNA damage (Kendall correlation test, τ = −0.24, FDR = 0.311). This finding was confirmed in the validation set [Wilcoxon test, 1590.08 (819.68–4816.81.68.81) and 1294.05 (934.45–2203.56.45.56), respectively, FDR > 0.05, Additional Fig. 4B]. MtDNA-CN correlated only with *POLD4* expression and showed no association with other selected DNA repair genes (see Additional Table 7), mtDNA damage (Kendall correlation test, τ = −0.06, FDR = 0.457), or patient characteristics such as sex, age, BMI, tumor location, TNM stage, tumor grade, or OS (all FDR > 0.05).

### MtDNA damage, mtDNA copy number, expression of DNA repair genes, and overall survival in the validation set

Multivariable analysis was conducted to assess the impact of mtDNA damage, mtDNA-CN, and the expression of selected DNA repair genes on the OS of patients in the validation set. None of these parameters showed a statistically significant association with OS.

### Comparison of mtDNA damage and mtDNA copy number between CC patients from the validation set and additional cohorts

**Fig. 3:**
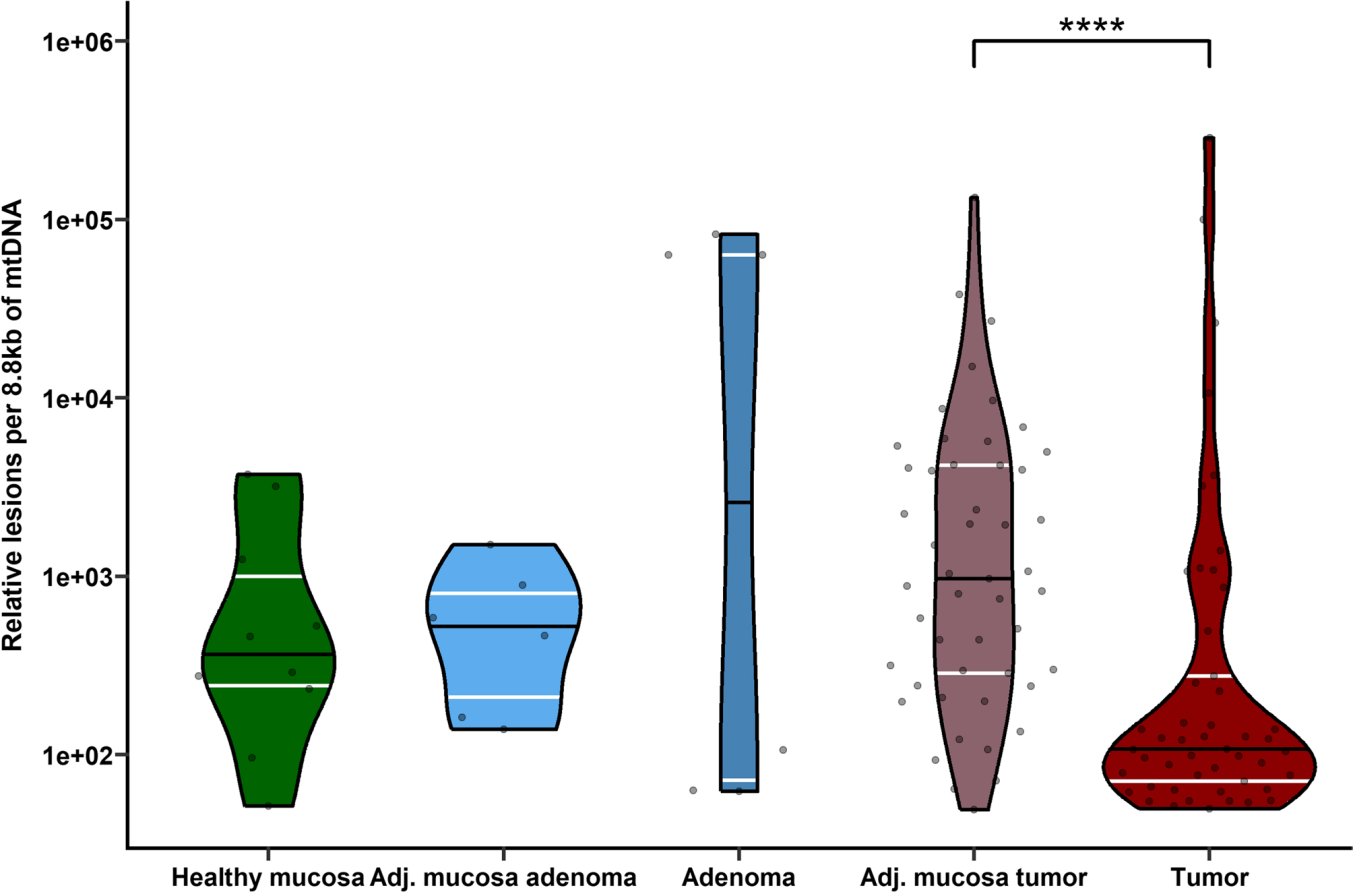
The extent of mtDNA damage across healthy individuals, patients with colon adenoma, and CC patients in the validation set. The figure shows the comparison of mtDNA damage levels in tissues from healthy control individuals (healthy mucosa), patients with colon adenomas (paired adjacent mucosa and adenoma), and CC patients (paired adjacent mucosa and tumor). The only significant difference was observed between tumors and their adjacent mucosa, with the latter exhibiting greater mtDNA damage. **** FDR < 0.0001. Abbreviations: Adj. mucosa – adjacent mucosa, mtDNA – mitochondrial DNA.

MtDNA damage and mtDNA-CN in tissues from CC patients (validation set) were compared with those from adenoma patients and healthy controls using the Wilcoxon test. A significant difference in mtDNA damage was observed only between tumors and their adjacent mucosa (Additional Figs. 2A and B), with higher damage in the latter [107.63 (70.97–276.91.97.91) vs. 969.88 (285.75–4190.04.75.04) respectively, FDR = 2.06e-05; see Fig. 3]. No correlation between mtDNA damage and mtDNA-CN was found in healthy individuals (Kendall correlation test, τ = −0.2, FDR = 0.74) or adenoma patients (Kendall correlation test, τ = 0.091, FDR = 0.74). MtDNA damage was also unrelated to adenoma patient characteristics (sex, age, adenoma type, location, and grade) and to control group characteristics (sex, age, BMI) (all FDR > 0.05).

**Fig. 4:**
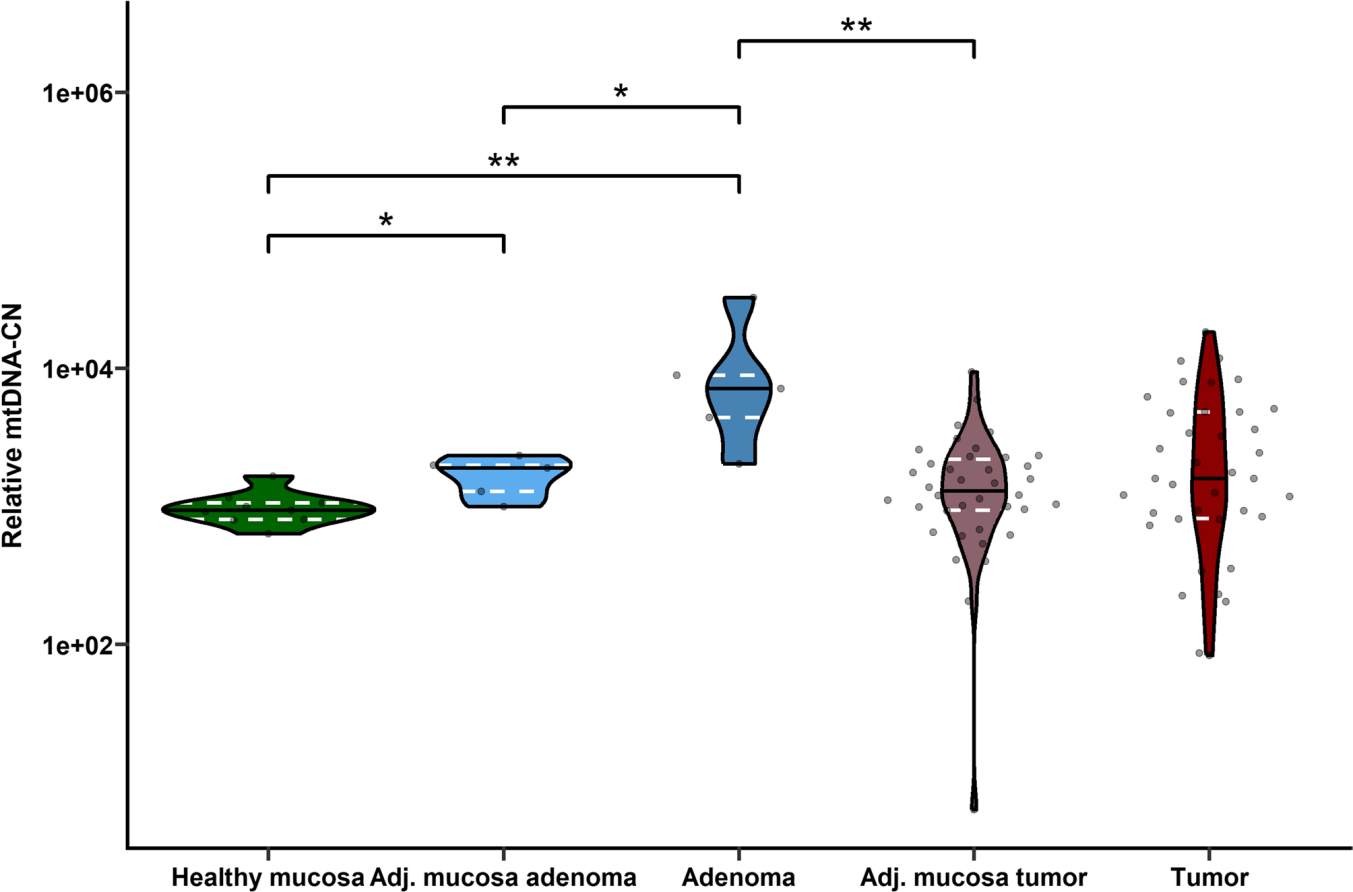
The mtDNA-CN levels across healthy individuals, patients with colon adenoma, and CC patients in the validation set. The figure compares mtDNA-CN levels in tissues from healthy control individuals (healthy mucosa), patients with colon adenomas (paired adjacent mucosa adenoma and adenoma), and CC patients (paired adjacent mucosa tumor and tumor). Colon adenomas displayed significantly higher mtDNA-CN compared to their adjacent non-affected mucosa. Both tissues showed increased mtDNA-CN compared to healthy control mucosa, tumor tissue, and tumor adjacent non-malignant mucosa. ** FDR < 0.01, *** FDR < 0.001. Abbreviations: Adj. mucosa – adjacent, mtDNA-CN – mitochondrial DNA copy number.

Colon adenomas exhibited significantly higher mtDNA-CN compared to adenoma-adjacent mucosa [7111.97 (4390.91–8881.61.91.61) and 1901.59 (1280.40–1990.38.40.38), respectively, FDR = 0.04, Fig. 4], healthy mucosa [7111.97 (4390.91–8881.61.91.61) and 940.53 (805.55–1058.17.55.17), respectively, FDR = 0.005, Fig. 4], and tumor-adjacent non-malignant mucosa [Wilcoxon test, 7111.97 (4390.91–8881.61.91.61) and 1294.05 (934.45–2203.56.45.56), respectively, FDR = 0.005, Fig. 4]. Adenoma-adjacent mucosa also had increased mtDNA-CN compared to healthy mucosa [1901.59 (1280.40–1990.38.40.38) and 940.53 (805.55–1058.17.55.17), respectively, FDR = 0.04, Fig. 4]. No differences in mtDNA-CN were detected between tumors and their adjacent non-malignant mucosa in either the pilot or validation sets [1590.08 (819.68–4816.81.68.81) vs. 1294.05 (934.45–2203.56.45.56), respectively, FDR > 0.05, as illustrated in Fig. 4], nor when compared tumors and their adjacent non-malignant mucosa in the validation set to healthy mucosa [1590.08 (819.68–4816.81.68.81) vs. 940.53 (805.55–1058.17.55.17), 1294.05 (934.45–2203.56.45.56) vs. 940.53 (805.55–1058.17.55.17), respectively, both FDR > 0.05, Fig. 4]. MtDNA-CN showed no association with adenoma patient characteristics (sex, age, adenoma type, location, grade) or control group characteristics (sex, age, BMI) (all FDR > 0.05).

### The expression and mutation frequency of DNA repair genes linked to mtDNA damage in the pilot set of CC patients and two additional cohorts

**Fig. 5:**
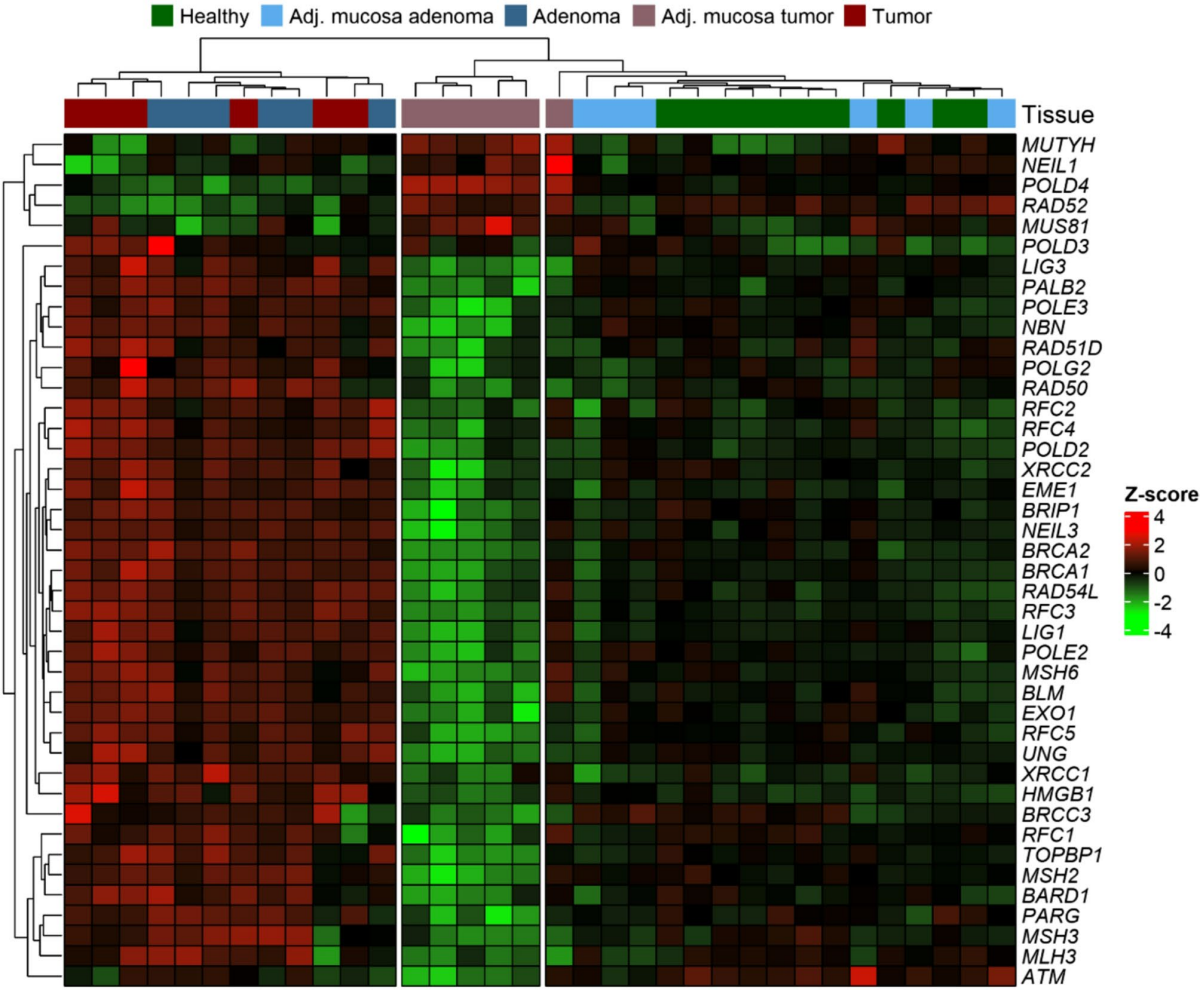
The expression of DNA repair genes associated with mtDNA damage in healthy individuals, adenoma patients, and CC patients (pilot set). The figure shows the DNA repair gene expression levels which were linked to mtDNA damage. Most of these genes were found to be overexpressed in tumors and adenomas when compared to the surrounding tissue (tumor- or adenoma-adjacent mucosa) and healthy tissue.

DNA repair gene expression was analyzed in healthy controls and colon adenoma patients by mRNA-Seq, with the results integrated alongside the data on CC patients from the pilot set. Most DNA repair genes associated with mtDNA damage were upregulated in both disease-affected tissues (tumors and adenomas) compared to tumor/adenoma-adjacent tissues and healthy tissues. Furthermore, the differences between tumors and tumor-adjacent mucosa were further accentuated by the concurrent downregulation of most DNA repair genes in the tumor-adjacent mucosa when compared to healthy tissue. However, expression levels in healthy tissues and adenoma-adjacent mucosa were comparable, showing no significant differences. Expression results are presented in the heatmap (Fig. 5) using Z-score transformation.

DNA repair genes were also analyzed for exon mutations based on RNAseq data in the pilot set of six CC and six adenoma patients. Mutations were detected in 18 out of 43 candidate DNA repair genes and included primarily frameshift variants followed by missense variants. The highest variant frequencies in adenomas and carcinomas were observed in *MSH6* (two different frameshift variants and one missense variant in 50% of patients – 5/6 tumors and 1/6 adenomas), *TOPBP1* (five different missense variants in 42% of patients – 2/6 tumors and 3/6 adenomas), and *NEIL1* (one frameshift variant, three different missense variants, and one splice acceptor variant in 42% of patients – 2/6 tumors and 3/6 adenomas), see Additional Table 8. A summary of the gene mutations, ranked by frequency, is visualized in the Oncoprint plot (Additional Fig. 5).

### The mitochondrial genes encoded by mitochondrial and nuclear genome and the association of their expression with mtDNA damage and mtDNA copy number in the pilot set

Expression of both mitochondrially encoded and nuclear-encoded mitochondrial genes was analyzed for associations with mtDNA damage and mtDNA-CN. Detailed results, including the differentially expressed genes and correlation analyses, are provided in Additional Tables 9, 10 and 11.

## Discussion

In the current study, we comprehensively examined, for the first time, mtDNA alterations and their potential role in CC etiology by analyzing mtDNA-CN, mtDNA damage, and the expression of candidate DNA repair genes from pathways with a confirmed or likely mitochondrial role. These parameters were compared across tissues from healthy controls, patients with precancerous lesions, and CC patients.

We assessed the damage in an 8.8 kb mtDNA region, finding significantly less damage in tumor tissue compared to the adjacent mucosa, consistent with Ericson et al. (Ericson et al. 2012)., who reported fewer mtDNA substitutions in CRC tissue. This lower mtDNA mutation burden in tumors may reflect a metabolic shift from oxidative phosphorylation to glycolysis (Ericson et al. 2012). Although one study found more mtDNA damage in rectal versus colon cancer (Potenza et al. 2011), most studies have focused on specific mtDNA mutations rather than complex mtDNA damage. In our study, no differences were observed when comparing tumor and adjacent mucosa with tissues from healthy controls and patients with colon adenomas, suggesting mtDNA damage may not be an early marker for CC development.

We then investigated the expression of DNA repair genes whose products are likely involved in mtDNA repair and might be linked to mtDNA damage. In the pilot set of CC patients, 43 DNA repair genes correlated with mtDNA damage, with 37 overexpressed in tumor tissues, suggesting that tumors may upregulate these genes to compensate for mtDNA damage. While mtDNA repair mechanisms remain incompletely understood, nDNA repair is well characterized. Inefficient DNA repair in non-tumor cells may promote carcinogenesis by the accumulation of DNA lesions and mutations, while increased DNA repair activity in tumors compared to tumor-adjacent tissue may support metastasis and chemotherapy resistance (Vodenkova et al. 2018). These possibilities are intriguing but remain speculative without functional validation, and further studies are required to determine whether the observed DNA repair activity directly drives disease aggressiveness. Overexpression of DNA repair genes has been observed in several malignancies, including CC (Chae et al. 2016). Interestingly, *NEIL1*,* RAD52*, and *POLD4* showed overexpression in the adjacent mucosa compared to tumor tissue. Due to their moderate to strong correlation with mtDNA damage, *NEIL1* and *POLD4* were, among others, selected for further validation, confirming their upregulation in adjacent mucosa and association with mtDNA damage.

Compared to healthy individuals and adenoma patients, most DNA repair genes were overexpressed in tumors and adenomas relative to their tumor-adjacent/adenoma-adjacent mucosa and healthy tissue. These changes appear to be influenced by the tumor microenvironment, which can modify the molecular phenotype of tumor-adjacent mucosa (Pietras and Ostman 2010; Sanz-Pamplona et al. 2014), potentially fostering pro-tumor or anti-tumor responses (Egeblad et al. 2010). Cancer cells can also induce molecular shifts in adjacent mucosa, facilitating malignant transformation (Egeblad et al. 2010). Exon mutations were identified in 18 of the 43 DNA repair genes across 6 adenoma and 6 CC patients. *MSH6* was the most frequently mutated, followed by *TOPBP1* and *NEIL1*. *MSH6* mutations are linked to Lynch syndrome, a hereditary predisposition to CRC, but also occurs in ~ 6% of sporadic CRC (Zhao et al. 2009). *TOPBP1* mutations were found in 3.0% of microsatellite instability (MSI)-high tumors (Kim et al. 2010). *NEIL1*, a BER-initiating glycosylase, has been associated with mutation burden in human cancers when underexpressed (Shinmura et al. 2016). This aligns with our results, as *NEIL1* was one of only three out of 43 genes downregulated in CC tumors.

In the validation phase with CC patients, we examined 12 genes that were moderately to strongly correlated with mtDNA damage and significantly differentially expressed between tumors and adjacent mucosa in the pilot set. Eleven genes remained correlated with mtDNA damage (*BRCA1*, *BRCA2*, *LIG1*, *LIG3*, *MRE11*, *NEIL1*, *NEIL3*, *POLD4*, *RAD50*, *RFC3*, and *UNG*, except for *PARG*), and all 12 showed significantly different expression between tumors and adjacent mucosa. Most genes, including *LIG3*,* NEIL1*,* NEIL3*,* PARG*,* POLD4*,* RFC3*,* and UNG*, are involved in the BER pathway, the predominant DNA repair pathway in mitochondria, and were overexpressed in tumors compared to adjacent mucosa, except for *NEIL1*, which was downregulated. This suggests that tumors may enhance DNA repair activity to manage mtDNA damage, similar to a study at the nDNA level, linking BER gene upregulation to sporadic CRC tumor aggressiveness (Leguisamo et al. 2017). Five genes involved in the HR pathway (*BRCA1*,* BRCA2*,* MRE11*,* POLD4*, and *RAD50*) were also overexpressed in tumors. HR gene mutations have been proposed as prognostic markers for CRC patients undergoing chemotherapy (Lin et al. 2022), with *MRE11* overexpression linked to poor OS in right-sided CRC (Ho et al. 2023; Naccarati et al. 2016). All the studied HR genes except for *POLD4* are also involved in NHEJ, another DNA repair pathway possibly active in mitochondria. Within the MMR pathway, whose deficiency is commonly associated with CRC (Taieb et al. 2022), *LIG1* and *RFC3* were overexpressed in tumors, while *POLD4* was upregulated in adjacent mucosa.

We also evaluated mitochondrially-encoded gene expression, which was 1.55–3.14.55.14-fold reduced in tumors versus adjacent non-tumor mucosa in the pilot set. The expression of eight genes (*MTATP6*,* MTCO1*,* MTCO3*,* MTND3*,* MTND5*, and *MTND6*) correlated with mtDNA damage, suggesting a functional link. This aligns with previous research showing increased expression of mtDNA protein-coding genes from adenomas to adenocarcinomas, likely driven by mtDNA mutations or post-transcriptional changes contributing to CRC progression (Wallace et al. 2016). Nineteen of the nuclear-encoded genes whose protein products are localized to and function within mitochondria, correlated with mtDNA damage and mtDNA-CN, indicating roles in oxidative phosphorylation or mitochondrial homeostasis (Boland et al. 2013). These findings highlight the complex interplay between mitochondrial and nuclear genomic alterations in CRC, as mitochondrial dysfunction has been shown to alter nuclear gene expression and induce genomic instability through retrograde signaling to the nucleus (Boland et al. 2013).

Another parameter evaluated in our study, mtDNA-CN, representing the total number of mtDNA molecules per cell, was higher in adenomas and their adjacent mucosa compared to mucosa from healthy individuals. Moreover, adenomas also showed higher mtDNA-CN than their adjacent mucosa and tumor-adjacent mucosa from CC patients. These results imply that mtDNA-CN reflects tissue energy needs rather than malignant transformation/state per se (Filograna et al. 2021; Reznik et al. 2016). Previous studies have reported both increased and decreased mtDNA-CN in CRC, with lower levels associated with *BRAF* mutations and MSI, and higher levels with *KRAS* mutations (Osch et al. 2015). Although some studies have found a connection between mtDNA-CN and CRC prognosis, results have been inconsistent. For example, correlations between mtDNA-CN and prognosis in MMR-deficient patients at stage II and III (Chen et al. 2024), as well as reduced mtDNA-CN levels in patients without neoadjuvant therapy who had postoperative complications, and in those with neoadjuvant therapy who did not (Potenza et al. 2011). However, we found no association between tissue mtDNA-CN and OS in CC patients in the validation set. This may be attributed to patient selection, tumor heterogeneity, or a metabolic shift to anaerobic glycolysis during the adenoma-to-carcinoma transition (Kim and Dang 2006).

The mtDNA-CN changes identified at the adenoma stage may reflect early mitochondrial stress responses. Importantly, distinguishing between adenomas that display mtDNA-CN increases and those that do not could provide a means of stratifying lesions with different malignant potential. In parallel, the deregulated expression of DNA repair genes observed in adenomas highlights a mechanistic link between impaired DNA maintenance and tumor initiation. Together, these findings indicate that mtDNA and DNA repair gene profiles may be integrated into molecular panels to improve early detection and risk stratification of colon neoplasia. The observed patterns may reflect adaptive or secondary responses to genomic instability. However, future studies employing mechanistic approaches will be necessary to clarify whether these alterations play a causal role in adenoma–carcinoma progression.

In summary, we analyzed a unique set of tissues from three cohorts: healthy controls, patients with colon adenomas, and CC patients, to investigate mtDNA changes in colon carcinogenesis. We evaluated three key parameters: mtDNA-CN, mtDNA damage, and the expression of DNA repair genes, and examined their interrelationships. Our findings revealed that mtDNA-CN was elevated in colon adenomas compared to healthy mucosa, adenoma-adjacent mucosa, and tumor-adjacent mucosa, with no difference observed between tumors and their adjacent mucosa. MtDNA-CN was also elevated in adenoma-adjacent mucosa compared to healthy mucosa. In contrast, mtDNA damage was significantly higher in tumor-adjacent mucosa than in tumor tissue, with no significant differences among other tissue types. Additionally, the expression of 11 out of 12 candidate DNA repair genes correlated with mtDNA damage and was significantly higher in tumors compared to the adjacent mucosa. A limitation of our study is the lack of formal, pathologist-estimated tumor cellularity for the fresh-frozen samples. Tissue acquisition was based on macroscopic evaluation by the operating surgeon, who selected viable tumor tissue while minimizing stromal or necrotic areas. Nevertheless, variable stromal and inflammatory cell content cannot be entirely excluded, and this may influence mtDNA-CN, mtDNA damage, and gene expression readouts. Future studies incorporating systematic histological review or expression-based purity estimates will be needed to more precisely account for tumor purity.

This study proposes several mtDNA-based biomarkers potentially involved in CC etiology and is among the first to comprehensively evaluate mtDNA-CN, mtDNA damage, and DNA repair gene expression across multiple tissue types. By integrating these parameters, we provide new insights into the potential role of mitochondrial alterations in early carcinogenic changes. While our findings support an association between mtDNA instability, DNA repair mechanisms, and colon neoplasia, further prospective and functional studies are needed to establish whether these processes actively drive tumor progression. Nonetheless, these observations indicate promising candidates for future biomarker development.

## Supplementary Information

Supplementary Material 1.

## Data Availability

The mtDNA data that support the findings of this study are available in Zenodo (https://handle.test.datacite.org/10.5072/zenodo.346173).

## Abbreviations

BER: Base excision repair
BMI: Body mass index
CC: Colon cancer
cDNA: Complementary DNA
CI: Confidence interval
CRC: Colorectal cancer
DSB: Double-strand breaks
DR: Direct reversal
FDR: False discovery rate
HR: Homologous recombination
HR: Hazard ratio
MMR: Mismatch repair
MSS: Microsatellite-stable
mtDNA: Mitochondrial DNA
mtDNA-CN: Mitochondrial DNA copy number
MT-ND1: Mitochondrial gene NADH dehydrogenase 1
mRNA-Seq: Messenger RNA sequencing
MSI: Microsatellite instability
nDNA: Nuclear DNA
NER: Nucleotide excision repair
NHEJ: Non-homologous end joining
OS: Overall survival
qPCR: Quantitative polymerase chain reaction
RIN: RNA integrity number
ROS: Reactive oxygen species
RT-qPCR: Reverse transcription qPCR
SSB: Single-strand breaks
TFAM: Mitochondrial transcription factor A
TNM: Tumor-node-metastasis
