# Supplementary material for: Dysregulated mitochondrial homeostasis and DNA repair in the progression from colon adenoma to cancer
Source: Mol Med. 2025 Nov 22;31:341. doi: 10.1186/s10020-025-01400-5 (PMC12751573; doi:10.1186/s10020-025-01400-5)
Supplement: Supplementary file 1 — Supplementary Material 1. [file 10020_2025_1400_MOESM1_ESM.docx]

Dysregulated Mitochondrial Homeostasis and DNA Repair in the Progression from Colon Adenoma to Cancer

Natalie Danesova^1,2^, Josef Horak^1,3^, Anna Valickova^1,4^, Adrian Gil-Korilis^1^, Jorge Ergui-Arbizu^1^, Richard Palek^5^, Jan Bruha^5^, Miroslav Levy^6^, Pavel Skrobanek^7^, Jan Kral^8,9^, Jiri Jungwirth^10,11^, Jiri Neuzil^12,13,14,15^, Veronika Vymetalkova^1^, Pavel Vodicka^1,2,4^, Sona Vodenkova^1,2*^, Kristyna Tomasova^1,2^

^1^Department of Molecular Biology of Cancer, Institute of Experimental Medicine of the Czech Academy of Sciences, Prague, Czech Republic

^2^Biomedical Centre, Faculty of Medicine in Pilsen, Charles University, Pilsen, Czech Republic

^3^Molecular Targets Program, Center for Cancer Research (CCR), National Cancer Institute (NCI), National Institutes of Health (NIH), Frederick, MD, USA

^4^Institute of Biology and Medical Genetics, First Faculty of Medicine, Charles University, Prague, Czech Republic

^5^Department of Surgery, Medical Faculty in Pilsen, Charles University, Pilsen, Czech Republic

^6^Department of Surgery, First Faculty of Medicine, Charles University and Thomayer Hospital, Prague, Czech Republic

^7^Department of Oncology, First Faculty of Medicine, Charles University and Thomayer Hospital, Prague, Czech Republic

^8^Department of Internal Medicine, University Hospital Motol, Second Faculty of Medicine, Charles University, Prague, Czech Republic

^9^Department of Hepatogastroenterology, Institute for Clinical and Experimental Medicine, Prague, Czech Republic

^10^Institute of Physiology, First Faculty of Medicine, Charles University, Prague, Czech Republic

^11^Department of Gastroenterology, Libera Scientia, Prague, Czech Republic

^12^Laboratory of Molecular Therapy, Institute of Biotechnology, Czech Academy of Sciences, Prague-West, Czech Republic

^13^Department of Genetics and Microbiology & Department of Physiology, Faculty of Science, Charles University, Prague, Czech Republic

^14^Department of Paediatrics and Inherited Metabolic Disorders, First Faculty of Medicine, Charles University, Prague, Czech Republic

^15^School of Pharmacy and Medical Science, Griffith University, Southport, Qld, Australia

* Corresponding author

**Key words:** colorectal cancer, colon adenomas, biomarkers, mitochondria, mitochondrial DNA damage, mitochondrial DNA repair, mitochondrial DNA copy number

**Corresponding author:** Email: sona.vodenkova@iem.cas.cz, Tel: +420 241062251, Full postal address: Department of Molecular Biology of Cancer, Institute of Experimental Medicine of the Czech Academy of Sciences, Videnska 1083, 142 00 Prague, Czech Republic. ORCID: 0000-0003-0315-5668.

# Additional Material
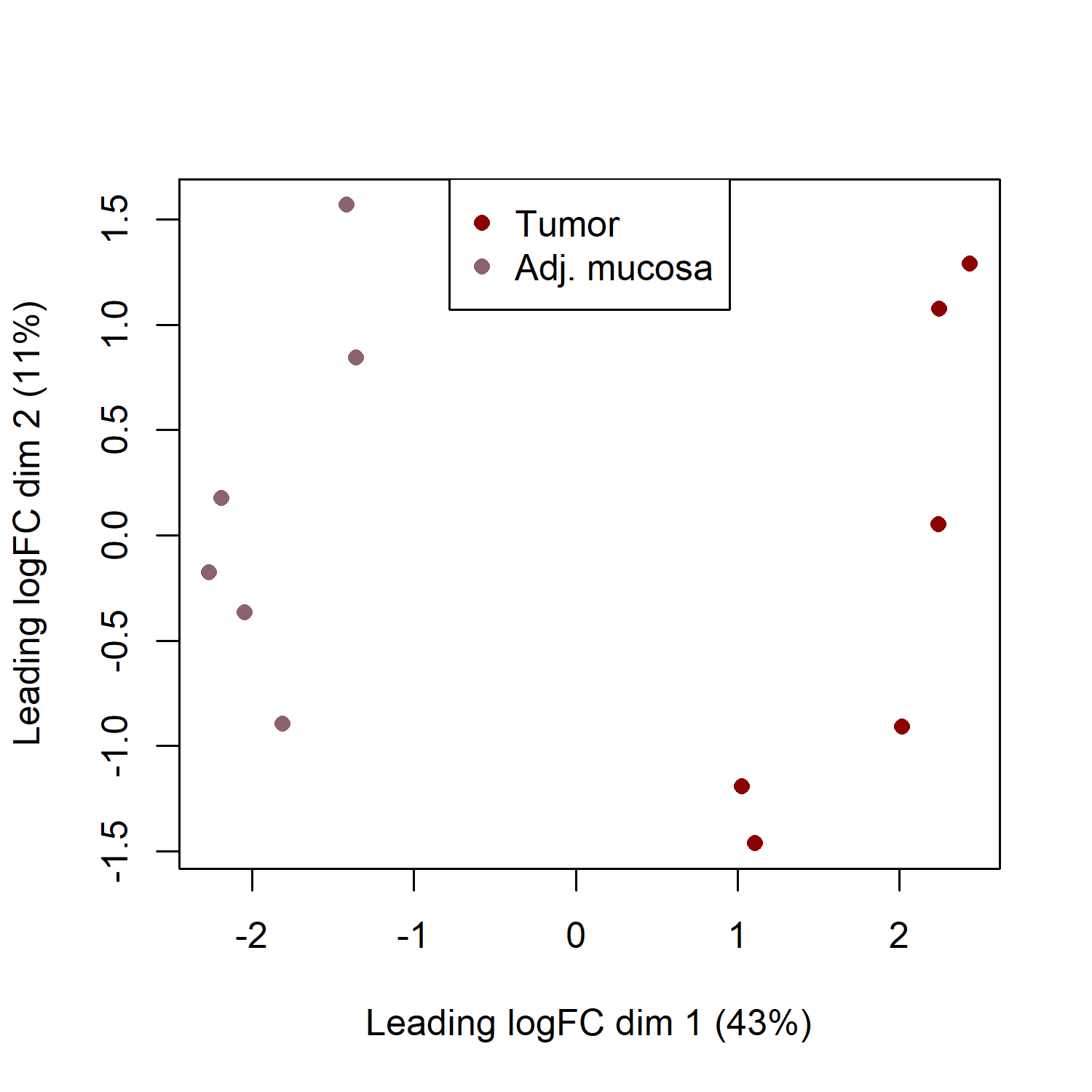


**Additional figure 1. MDS plot showing the separation between tumor and adjacent mucosa samples**. The figure shows two-dimensional MDS plot illustrating the clustering of tumor (red) and adjacent mucosa (purple) samples.

Abbreviations: Adj. mucosa – adjacent mucosa


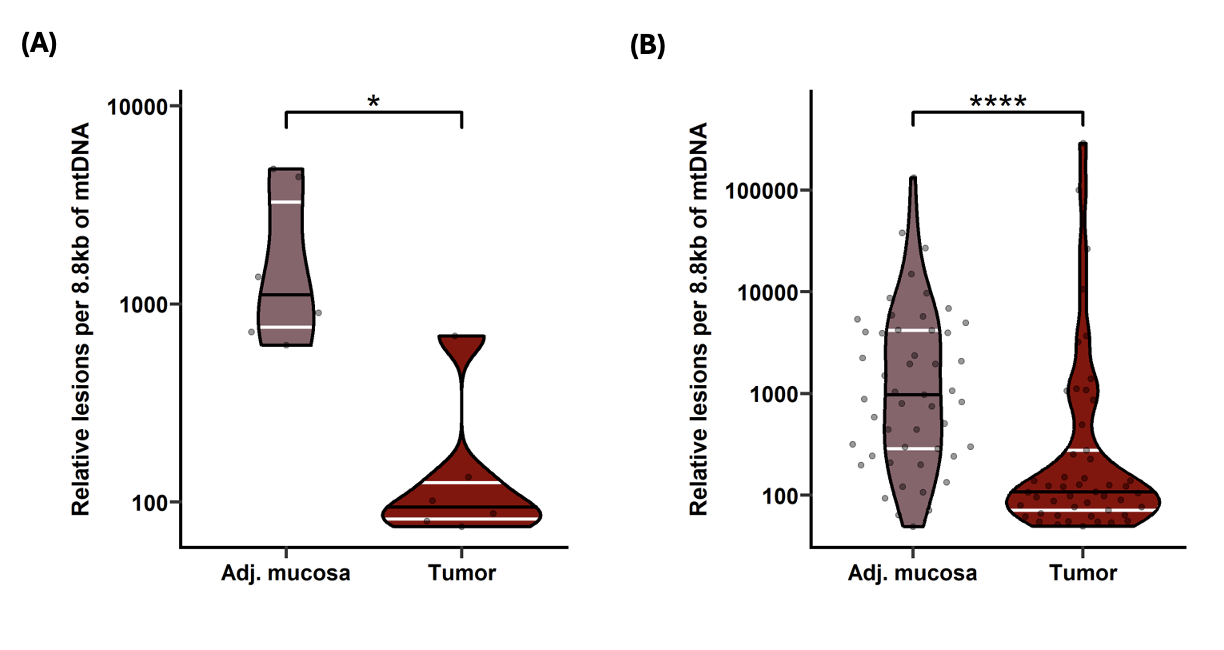
**Additional Figure 2. Extent of mtDNA damage in tumors and adjacent mucosa in the pilot set (A) and validation set (B).** The figures show that adjacent mucosa exhibits more mtDNA damage than tumors in both patient sample sets. * FDR < 0.05, **** FDR < 0.0001.

Abbreviations: Adj. mucosa – adjacent mucosa, mtDNA – mitochondrial DNA.


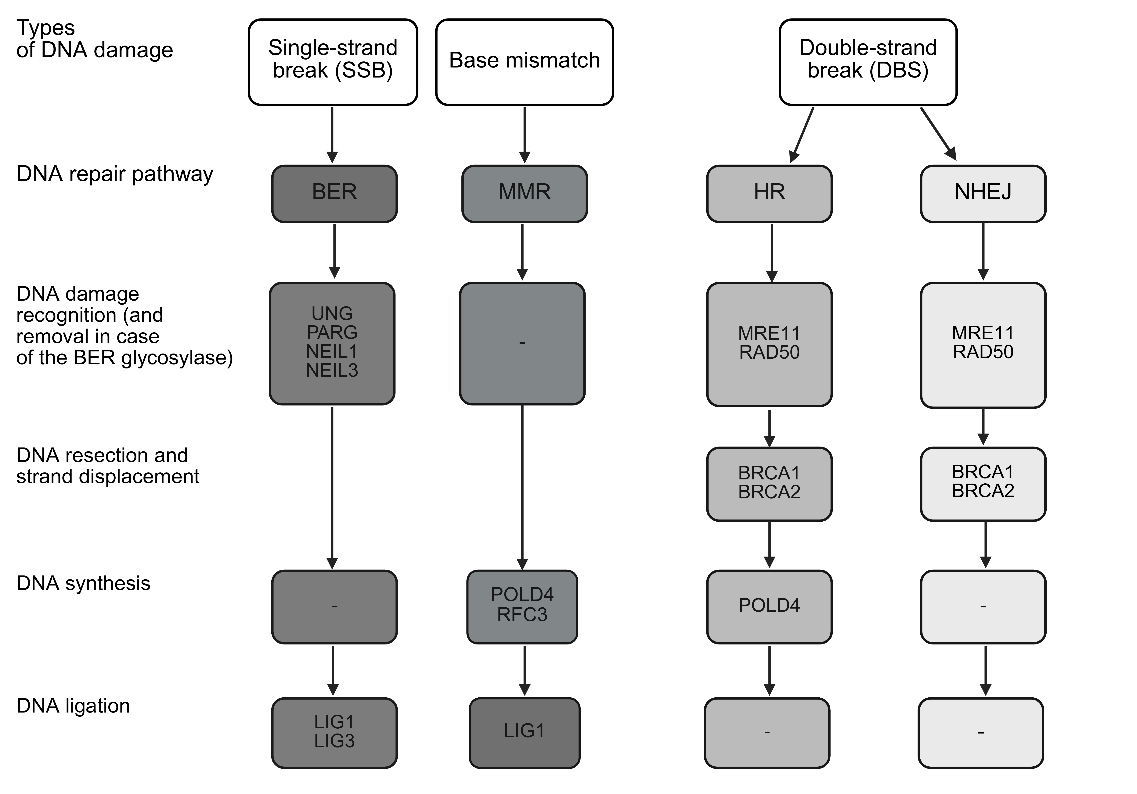


**Additional Figure 3. Twelve DNA repair genes selected for the validation study, showing their role in specific DNA repair pathways.** These genes were selected based on the moderate to strong correlation (correlation coeficient≥0.6) between their expression levels and mtDNA damage, as well as their significantly different expression profiles between tumors and adjacent mucosa observed in the pilot set. These selected genes are involved in pathways responsible for the repair of single-strand breaks, double-strand breaks, and base mismatches. They primarily include glycosylases, polymerases, and ligases.

Abbreviations: BER – base excision repair, HR – homologous recombination, MMR – mismatch repair, mtDNA -mitochondrial DNA, NHEJ – non-homologous end joining.


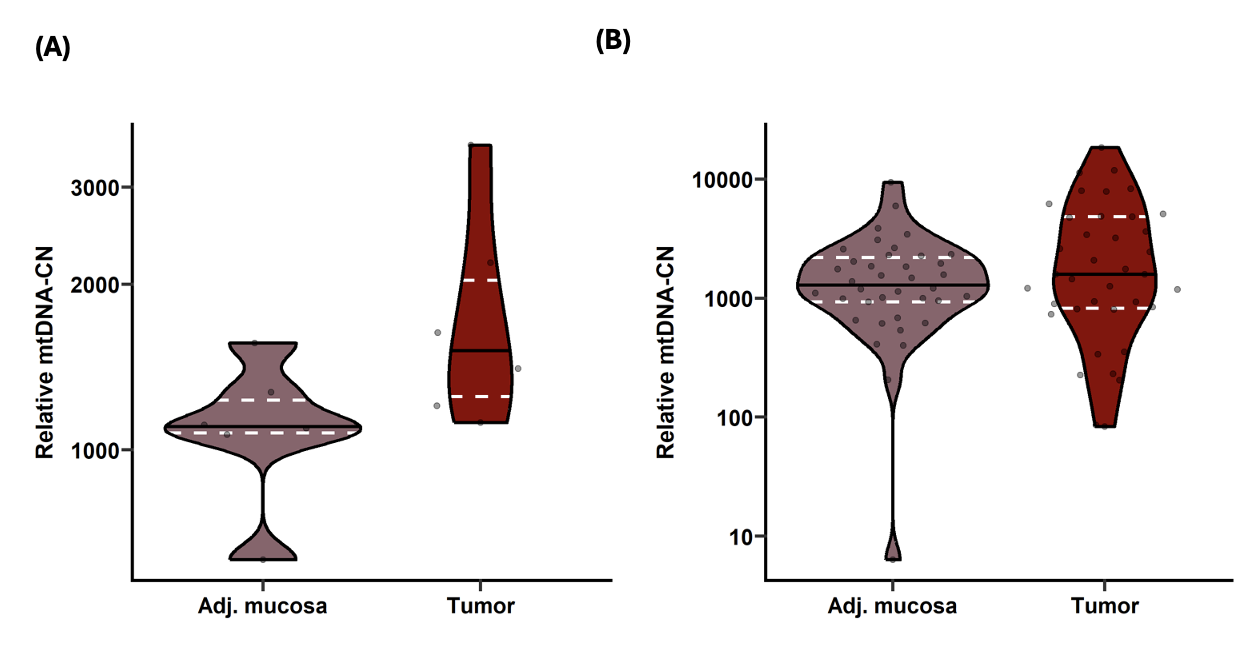
**Additional Figure 4. Amount of mtDNA-CN in tumors and adjacent mucosa in the pilot set (A) and validation set (B)**. The figures illustrate mtDNA-CN in tumors compared to adjacent mucosa, showing no difference between the two tissues.

Abbreviations: Adj. mucosa – adjacent mucosa, mtDNA-CN – mitochondrial DNA copy number.

**
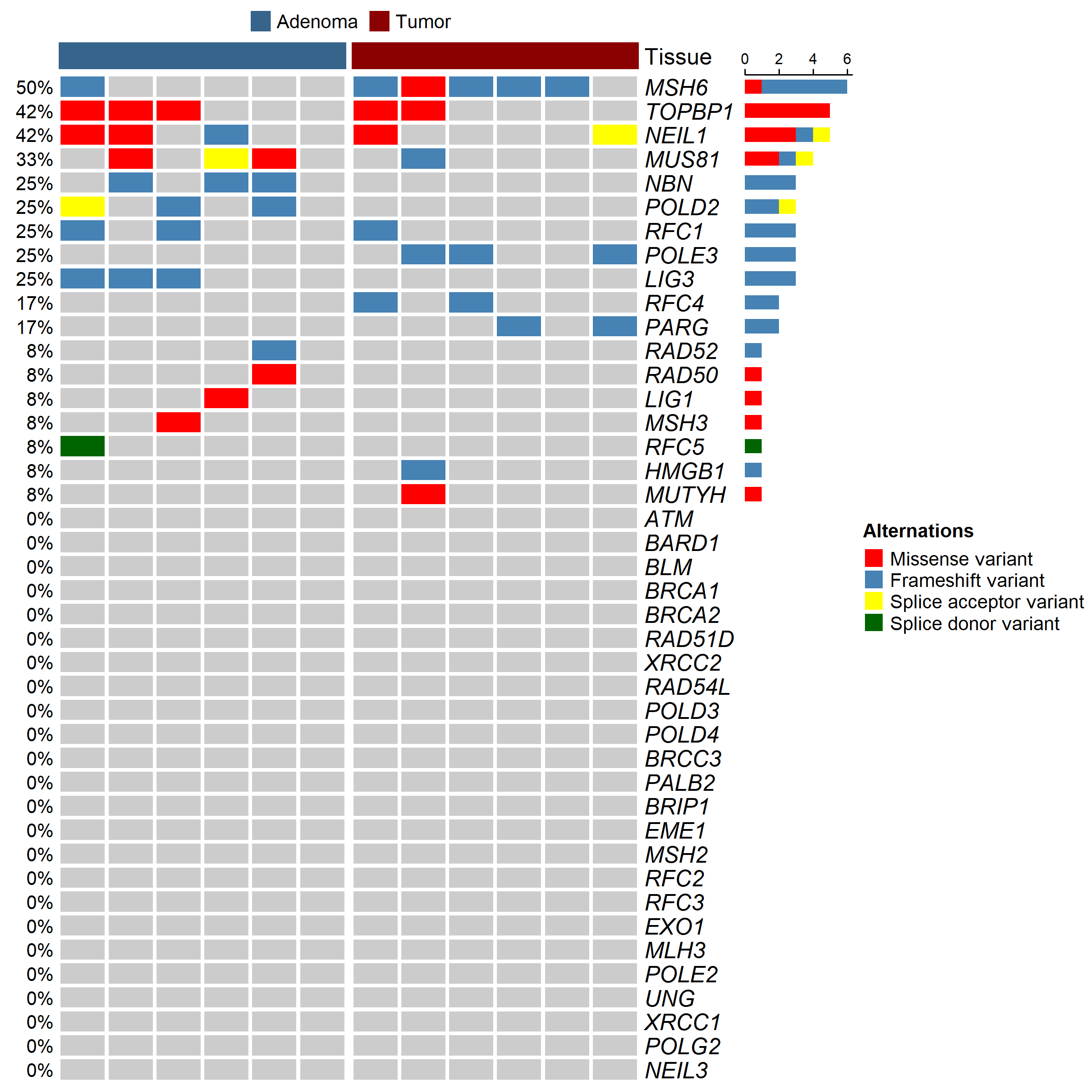
Additional** **Figure 5. Oncoprint plot showing types of mutations in DNA repair genes found in adenoma and tumor samples from colon adenoma patients and CC patients included in the pilot set.** The figure shows the frequency and types of mutations in 43 selected DNA repair genes in both adenomas and tumors.

Abbreviations: CC – colon cancer

**Additional Table 1**. RNA integrity (RIN) numbers for RNA samples used for RNA sequencing library preparation.

| **1A)** | **Sample ID** | **RNA integrity number (RIN)** |  |  |  |  |  |  |  |
| --- | --- | --- | --- | --- | --- | --- | --- | --- | --- |
|  | V01T | 9.4 |  |  |  |  |  |  |  |
|  | V01H | 6.4 |  |  |  |  |  |  |  |
|  | V04T | 7.5 |  |  |  |  |  |  |  |
|  | V04H | 6.8 |  |  |  |  |  |  |  |
|  | V05T | 5.3 |  |  |  |  |  |  |  |
|  | V05H | 6.7 |  |  |  |  |  |  |  |
|  | V06T | 8.4 |  |  |  |  |  |  |  |
|  | V06H | 7.4 |  |  |  |  |  |  |  |
|  | V09T | 8.3 |  |  |  |  |  |  |  |
|  | V09H | 6.0 |  |  |  |  |  |  |  |
|  | V10T | 7.7 |  |  |  |  |  |  |  |
|  | V10H | 5.6 |  |  |  |  |  |  |  |
|  |  |  |  |  |  |  |  |  |  |
| **1B)** | **Sample ID** | **RNA integrity number (RIN)** |  |  |  |  |  |  |  |
|  | J28 | 9.2 |  |  |  |  |  |  |  |
|  | J63 | 8.7 |  |  |  |  |  |  |  |
|  | J65 | 9.3 |  |  |  |  |  |  |  |
|  | J79 | 9.1 |  |  |  |  |  |  |  |
|  | J90 | 8.7 |  |  |  |  |  |  |  |
|  | J97 | 8.3 |  |  |  |  |  |  |  |
|  |  |  |  |  |  |  |  |  |  |
| **1C)** | **Sample ID** | **RNA integrity number (RIN)** |  |  |  |  |  |  |  |
|  | J02Z | 9.6 |  |  |  |  |  |  |  |
|  | J03Z | 9.0 |  |  |  |  |  |  |  |
|  | J04Z | 8.9 |  |  |  |  |  |  |  |
|  | J05Z | 9.4 |  |  |  |  |  |  |  |
|  | J06Z | 9.7 |  |  |  |  |  |  |  |
|  | J07Z | 9.0 |  |  |  |  |  |  |  |
|  | J08Z | 9.0 |  |  |  |  |  |  |  |
|  | J09Z | 9.3 |  |  |  |  |  |  |  |
|  | J10Z | 9.5 |  |  |  |  |  |  |  |
|  | H003 | 8.8 |  |  |  |  |  |  |  |

Abbreviation: RIN = RNA integrity number.

**Additional Table 2**. TaqMan Assays used for gene expression quantification in the validation set.

| **Gene name** | **Type of gene** | **Assay number** | **Color** |  |  |  |  |  |  |
| --- | --- | --- | --- | --- | --- | --- | --- | --- | --- |
| *BRCA1* | Repair | Hs01556193_m1 | FAM |  |  |  |  |  |  |
| *BRCA2* | Repair | Hs00609073_m1 | FAM |  |  |  |  |  |  |
| *LIG1* | Repair | Hs01553527_m1 | FAM |  |  |  |  |  |  |
| *LIG3* | Repair | Hs00242692_m1 | FAM |  |  |  |  |  |  |
| *MRE11* | Repair | Hs00967437_m1 | FAM |  |  |  |  |  |  |
| *NEIL1* | Repair | Hs00908563_m1 | FAM |  |  |  |  |  |  |
| *NEIL3* | Repair | Hs00217387_m1 | FAM |  |  |  |  |  |  |
| *PARG* | Repair | Hs00608254_m1 | FAM |  |  |  |  |  |  |
| *POLD4* | Repair | Hs01087022_g1 | FAM |  |  |  |  |  |  |
| *RAD50* | Repair | Hs00990023_m1 | FAM |  |  |  |  |  |  |
| *RFC3* | Repair | Hs01082404_m1 | FAM |  |  |  |  |  |  |
| *UNG* | Repair | Hs01037093_m1 | FAM |  |  |  |  |  |  |
| *GAPDH* | Housekeeping | Hs02786624_g1 | FAM |  |  |  |  |  |  |
| *ACTB* | Housekeeping | Hs01060665_g1 | FAM |  |  |  |  |  |  |

**Additional Table 3**. Statistical values for all 43 DNA repair genes whose expression correlated with mtDNA damage in the pilot set.

| **Gene** | **Localization** | **DNA repair pathway** | **Spearman's Rho** | **P-Value FDR** |
| --- | --- | --- | --- | --- |
| *LIG3* | chromosome 17 | BER | -0.877 | **< 0.001 0.017** |
| *MUTYH* | chromosome 1 | BER | 0.824 | **< 0.001 0.017** |
| *BRIP1* | chromosome 17 | HR | -0.771 | **0.002 0.017** |
| *RAD50* | chromosome 5 | HR, NHEJ | -0.763 | **0.002 0.017** |
| *PALB2* | chromosome 16 | HR | -0.758 | **0.003 0.017** |
| *RAD52* | chromosome 12 | HR | 0.754 | **0.003 0.017** |
| *BRCA2* | chromosome 13 | HR, NHEJ | -0.745 | **0.003 0.017** |
| *BRCA1* | chromosome 17 | HR, NHEJ | -0.741 | **0.004 0.017** |
| *TOPBP1* | chromosome 3 | HR | -0.741 | **0.004 0.017** |
| *BARD1* | chromosome 2 | HR | -0.732 | **0.004 0.017** |
| *EXO1* | chromosome 1 | MMR | -0.719 | **0.005 0.017** |
| *BLM* | chromosome 15 | HR | -0.710 | **0.006 0.017** |
| *NBN* | chromosome 8 | HR | -0.710 | **0.006 0.017** |
| *BRCC3* | chromosome X | HR | -0.705 | **0.006 0.017** |
| *LIG1* | chromosome 19 | BER, MMR | -0.705 | **0.006 0.017** |
| *PARG* | chromosome 10 | BER | -0.688 | **0.008 0.021** |
| *RAD51D* | chromosome 17 | HR | -0.684 | **0.009 0.022** |
| *NEIL3* | chromosome 4 | BER | -0.679 | **0.010 0.022** |
| *XRCC2* | chromosome 7 | HR | -0.666 | **0.011 0.022** |
| *POLD3* | chromosome 11 | BER, HR, MMR | -0.662 | **0.012 0.022** |
| *MSH2* | chromosome 2 | MMR | -0.662 | **0.012 0.022** |
| *MSH3* | chromosome 5 | MMR | -0.662 | **0.012 0.022** |
| *POLD4* | chromosome 11 | BER, HR, MMR | 0.662 | **0.012 0.022** |
| *MRE11* | Chrosomosome 11 | HR | -0.657 | **0.013 0.022** |
| *RFC3* | chromosome 13 | BER, MMR | -0.657 | **0.013 0.022** |
| *MLH3* | chromosome 14 | MMR | -0.657 | **0.013 0.022** |
| *NEIL1* | chromosome 15 | BER | 0.644 | **0.015 0.024** |
| *UNG* | chromosome 12 | BER | -0.600 | **0.026 0.040** |
| *ATM* | chromosome 11 | HR | -0.596 | **0.028 0.041** |
| *MSH6* | chromosome 2 | MMR | -0.596 | **0.028 0.041** |
| *EME1* | chromosome 17 | HR | -0.587 | **0.030 0.041** |
| *POLE2* | chromosome 14 | BER | -0.587 | **0.030 0.041** |
| *RFC5* | chromosome 12 | BER, MMR | -0.578 | **0.033 0.043** |
| *RAD54L* | chromosome 7 | HR | -0.569 | **0.037 0.045** |
| *POLE3* | chromosome 9 | BER | -0.569 | **0.037 0.045** |
| *POLD2* | chromosome 7 | BER, HR, MMR | -0.560 | **0.040 0.045** |
| *RFC1* | chromosome 4 | BER, MMR | -0.560 | **0.040 0.045** |
| *XRCC1* | chromosome 19 | BER | -0.560 | **0.040 0.045** |
| *RFC4* | chromosome 3 | BER, MMR | -0.552 | **0.044 0.047** |
| *MUS81* | chromosome 11 | HR | 0.552 | **0.044 0.047** |
| *POLG2* | chromosome 17 | BER | -0.547 | **0.046 0.048** |
| *RFC2* | chromosome 7 | BER, MMR | -0.543 | **0.048 0.048** |
| *HMGB1* | chromosome 13 | BER | -0.543 | **0.048 0.048** |

Abbreviations: BER – base excision repair, HR – homologous recombination, mtDNA – mitochondrial DNA, MMR – mismatch repair, NHEJ – non-homologous recombination.

**Additional Table 4**. Statistical values for all 37 DNA repair genes whose expression correlated with mtDNA damage and had a different expression profile in tumors and adjacent mucosa in the pilot set.

| **Gene** | **Spearman's Rho** | **FDR** | **logFC** | **FDR** |
| --- | --- | --- | --- | --- |
| *NEIL1* | 0.644 | **0.024** | -1.130 | **2.47E-05** |
| *RAD52* | 0.754 | **0.017** | -0.805 | **0.003** |
| *POLD4* | 0.662 | **0.022** | -1.813 | **1.302E-17** |
| *MRE11* | -0.657 | **0.022** | 1.183 | **6.964E-07** |
| *POLE3* | -0.570 | **0.045** | 1.112 | **6.166E-08** |
| *NBN* | -0.710 | **0.017** | 1.110 | **3.082E-07** |
| *RAD51D* | -0.684 | **0.022** | 0.838 | **< 0.001** |
| *EME1* | -0.587 | **0.041** | 2.065 | **9.460E-10** |
| *XRCC2* | -0.666 | **0.022** | 2.948 | **2.815E-09** |
| *POLG2* | -0.547 | **0.048** | 0.969 | **< 0.001** |
| *RFC4* | -0.552 | **0.047** | 1.557 | **0.000** |
| *RFC2* | -0.543 | **0.048** | 1.222 | **1.044E-06** |
| *POLD2* | -0.560 | **0.045** | 1.895 | **9.210E-21** |
| *MSH6* | -0.596 | **0.041** | 1.283 | **< 0.001** |
| *BLM* | -0.710 | **0.017** | 1.757 | **< 0.001** |
| *POLE2* | -0.587 | **0.041** | 1.896 | **< 0.001** |
| *RFC3* | -0.657 | **0.022** | 2.477 | **2.309E-13** |
| *BRCA2* | -0.745 | **0.017** | 2.386 | **5.129E-11** |
| *LIG1* | -0.705 | **0.017** | 1.547 | **1.242E-06** |
| *NEIL3* | -0.679 | **0.022** | 3.175 | **< 0.001** |
| *RAD54L* | -0.569 | **0.045** | 2.608 | **1.530E-10** |
| *BARD1* | -0.732 | **0.017** | 1.240 | **0.001** |
| *BRCA1* | -0.741 | **0.017** | 2.050 | **1.877E-10** |
| *MSH2* | -0.662 | **0.022** | 1.482 | **< 0.001** |
| *BRIP1* | -0.771 | **0.017** | 2.613 | **3.249E-07** |
| *TOPBP1* | -0.741 | **0.017** | 0.973 | **< 0.001** |
| *RFC5* | -0.578 | **0.043** | 1.479 | **4.534E-08** |
| *PALB2* | -0.758 | **0.017** | 1.469 | **2.344E-08** |
| *EXO1* | -0.719 | **0.017** | 2.890 | **7.112E-09** |
| *UNG* | -0.600 | **0.040** | 1.456 | **1.241E-09** |
| *BRCC3* | -0.705 | **0.017** | 0.626 | **0.011** |
| *LIG3* | -0.877 | **0.017** | 1.086 | **8.485E-06** |
| *RAD50* | -0.763 | **0.017** | 0.780 | **< 0.001** |
| *RFC1* | -0.560 | **0.045** | 0.606 | **0.021** |
| *XRCC1* | -0.560 | **0.045** | 0.655 | **0.002** |
| *HMGB1* | -0.543 | **0.048** | 1.157 | **6.479E-07** |
| *PARG* | -0.688 | **0.021** | 0.565 | **0.005** |

Abbreviations: FC – fold change, mtDNA – mitochondrial DNA.

**Additional Table 5.** Selected DNA repair genes for the validation set and their correlation with mtDNA damage.

| **Gene** | **Spearman´s Rho** | **P-Value FDR** |
| --- | --- | --- |
| *BRCA1* | -0.229 | **0.021 0.025** |
| *BRCA2* | -0.293 | **0.003 0.009** |
| *LIG1* | -0.278 | **0.005 0.012** |
| *LIG3* | -0.263 | **0.008 0.016** |
| *MRE11* | -0.235 | **0.018 0.025** |
| *NEIL1* | 0.231 | **0.020 0.025** |
| *NEIL3* | -0.226 | **0.023 0.025** |
| *PARG* | -0.166 | 0.096 0.096 |
| *POLD4* | 0.336 | **0.001 0.006** |
| *RAD50* | -0.256 | **0.010 0.017** |
| *RFC3* | -0.329 | **0.001 0.006** |
| *UNG* | -0.297 | **0.003 0.009** |

Abbreviations: mtDNA – mitochondrial DNA.

**Additional Table 6.** Statistical values for the selected DNA repair genes for the validation set which had a different expression profile in tumors and their adjacent mucosa.

| **Gene** | **logFC** | **P-Value FDR** |
| --- | --- | --- |
| *BRCA1* | 3.32 | **9.05E-13 3.62E-12** |
| *BRCA2* | 5.92 | **1.22E-13 7.32E-13** |
| *LIG1* | 2.50 | **5.95E-12 1.79E-11** |
| *LIG3* | 1.82 | **3.80E-06 4.56E-06** |
| *MRE11* | 2.18 | **1.56E-10 3.74E-10** |
| *NEIL1* | 0.46 | **7.90E-06 8.51E-06** |
| *NEIL3* | 2.58 | **3.35E-10 5.74E-10** |
| *PARG* | 1.89 | **1.97E-08 2.63E-08** |
| *POLD4* | 0.55 | **8.51E-06 8.51E-06** |
| *RAD50* | 1.81 | **2.90E-10 5.74E-10** |
| *RFC3* | 5.68 | **9.19E-14 7.32E-13** |
| *UNG* | 3.02 | **2.97E-09 4.46E-09** |

Abbreviations: FC – fold change.

**Additional Table 7**. Selected DNA repair genes and their correlation with mtDNA-CN in the validation set.

| **Gene** | **Spearman´s Rho** | **P-Value FDR** |
| --- | --- | --- |
| *BRCA1* | -0.049 | 0.622 0.992 |
| *BRCA2* | -0.096 | 0.334 0.992 |
| *LIG1* | 0.012 | 0.902 0.992 |
| *LIG3* | -0.066 | 0.505 0.992 |
| *MRE11* | -0.065 | 0.509 0.992 |
| *NEIL1* | 0.001 | 0.992 0.992 |
| *NEIL3* | -0.086 | 0.388 0.992 |
| *PARG* | 0.020 | 0.839 0.992 |
| *POLD4* | -0.242 | **0.014** 0.168 |
| *RAD50* | 0.010 | 0.919 0.992 |
| *RFC3* | -0.017 | 0.861 0.992 |
| *UNG* | -0.055 | 0.581 0.992 |

Abbreviations: mtDNA-CN – mitochondrial DNA copy number.

**Additional Table 8.** Overview of mutations in selected DNA repair genes in colon adenomas and CC tumors.

| **Gene** | **Whole cohort (n = 12)** | **Localization** | **Position** | **Effect** | **Reference** | **Alternative** | **Number of mutated tumors (n = 6)** | **Number of mutated adenomas (n = 6)** |
| --- | --- | --- | --- | --- | --- | --- | --- | --- |
| *MSH6* | 6 (50%) | chromosome 2 | 47800667 | Frameshift variant | C | CA | 3 | 1 |
|  |  |  | 47803552 | Frameshift variant | C | CT | 3 | 0 |
|  |  |  | 47800600 | Missense variant | G | A | 1 | 0 |
| *TOPBP1* | 5 (42%) | chromosome 3 | 133601269 | Missense variant | T | G | 2 | 1 |
|  |  |  | 133617281 | Missense variant | T | A | 0 | 1 |
|  |  |  | 133617327 | Splice acceptor variant&intron variant | C | CCAG | 0 | 1 |
|  |  |  | 133618390 | Missense variant | G | T | 0 | 1 |
|  |  |  | 133601285 | Stop gained | C | A | 1 | 0 |
| *NEIL1* | 5 (42%) | chromosome 15 | 75352229 | Frameshift variant&splice acceptor variant&splice donor variant&splice region variant&intron variant | CGGTCAGCAAGCAGGCATGGGCATGGGGACTGCGGTGGGCCAGGTGTGCCCACATTCCCCACTGCCTAGCATGGCTTGCCTTGCCCCCACTACA | C | 0 | 2 |
|  |  |  | 75351340 | Missense variant | A | G | 1 | 1 |
|  |  |  | 75352083 | Missense variant | C | A | 0 | 1 |
|  |  |  | 75349321 | Splice acceptor variant&intron variant | A | G | 1 | 0 |
| *MUS81* | 4 (33%) | chromosome 11 | 65863718 | Splice acceptor variant&splice donor variant&conservative inframe deletion&splice region variant&intron variant | CCAGGTGAAGGGCCGTGGACAGGCTGGCACCAGGGGCAGGGCCTGGTGGGTAGGGGATCGCAAGCTAACGGCTGGCTTGTCAG | C | 0 | 1 |
|  |  |  | 65866039 | Missense variant | G | C | 0 | 1 |
|  |  |  | 65863833 | Missense variant | A | T | 0 | 1 |
|  |  |  | 65863501 | Frameshift variant&splice acceptor variant&splice donor variant&splice region variant&intron variant | GGGTGAGTGAGGTGGGGAGAAACGAGGGAGATGATCAGAGGAGGCTGGGGGGTAGGCACTGCCCTGCTCTGATCTAGGCTTCCCTCCTTGCCACTCCA | G | 1 | 0 |
| *NBN* | 3 (25%) | chromosome 8 | 89946251 | Frameshift variant | C | CT | 0 | 2 |
|  |  |  | 89953437 | Frameshift variant | C | CT | 0 | 1 |
| *POLD2* | 3 (25%) | chromosome 7 | 44117013 | Frameshift variant splice acceptor variant&splice region variant&intron variant | AACCTGCGGGAGAAGGTGGGGTCCTCCAGGGTGCCGAGAAGGGAGGCAGCCCCTCCTAGGTGCAACTCAAAGATTCCACTGACCATGAGCTGGTTCCAGCTCCCGAGAACTGCTGCTC | A | 0 | 2 |
|  |  |  | 44117248 | Splice acceptor variant&intron variant | C | CCGTAACCAGCTTTGACACGTCAATGGTGCCTTTTAGTTTGATACGCTGCAGTTCATCTTCCAAGACCAGCTCGAGA | 0 | 1 |
| *RFC1* | 3 (25%) | chromosome 4 | 39308991 | Frameshift variant | A | AT | 0 | 2 |
|  |  |  | 39308889 | Frameshift variant | C | CT | 1 | 0 |
| *POLE3* | 3 (25%) | chromosome 9 | 113408914 | Frameshift variant | G | GT | 3 | 0 |
| *LIG3* | 3 (25%) | chromosome 17 | 34983466 | Frameshift variant | C | CA | 0 | 3 |
| *RFC4* | 2 (17%) | chromosome 3 | 186790063 | Frameshift variant&splice acceptor variant&splice donor variant&splice region variant&intron variant | TCCTACGAGAAAAATTTAAGAAATTAGCATCCTTCAGGTAGTTAAATGTTCCATTGACATGTGCAAAGTACCTACTTA | T | 2 | 0 |
|  |  |  | 186790190 | Disruptive inframe insertion | T | TAGA | 1 | 0 |
| *PARG* | 2 (17%) | chromosome 10 | 49933617 | Frameshift variant | A | AT | 2 | 0 |
| *RAD52* | 1 (8%) | chromosome 12 | 916483 | Frameshift variant&splice acceptor variant&splice donor variant&splice region variant&intron variant | TCTGCATGAGAGGGCGGCGGCGAGGACGGGCTCCTGAGCAACAGCCGCGGCTGCTGGGAGGACACGCACGGCTGGCTGGCTCTGGAGGCCTGAGTGGAGGCAGCCCCGTGACACAGGAGGGGCCGCAGAGGAAAGGAGGGGACTTAGGCCGCATAC | T | 0 | 1 |
| *RAD50* | 1 (8%) | chromosome 5 | 132591321 | Missense variant | C | T | 0 | 1 |
| *LIG1* | 1 (8%) | chromosome 19 | 48137624 | Missense variant | C | A | 0 | 1 |
| *MSH3* | 1 (8%) | chromosome 5 | 80654825 | Missense variant | G | A | 0 | 1 |
| *RFC5* | 1 (8%) | chromosome 12 | 118016893 | Splice donor variant&intron variant | G | GGTTGAAAAA | 0 | 1 |
| *HMGB1* | 1 (8%) | chromosome 13 | 30463242 | Frameshift variant | C | CT | 1 | 0 |
| *MUTYH* | 1 (8%) | chromosome 1 | 45332656 | Missense variant | C | G | 1 | 0 |

**Additional Table 9**. Mitochondrially encoded protein-coding gene expressions in tumors compared to the adjacent mucosa.

| **Gene** | **logFC** | **FDR** |  |  |  |  |  |  |  |  |
| --- | --- | --- | --- | --- | --- | --- | --- | --- | --- | --- |
| *MT.ATP6* | -0.79 | **0.014** |  |  |  |  |  |  |  |  |
| *MT.ATP8* | -0.76 | **0.015** |  |  |  |  |  |  |  |  |
| *MT.CO1* | -1.33 | **1.09E-05** |  |  |  |  |  |  |  |  |
| *MT.CO2* | -0.86 | **0.004** |  |  |  |  |  |  |  |  |
| *MT.CO3* | -1.00 | **0.001** |  |  |  |  |  |  |  |  |
| *MT.CYB* | -0.98 | **0.002** |  |  |  |  |  |  |  |  |
| *MT.ND1* | -0.98 | **0.006** |  |  |  |  |  |  |  |  |
| *MT.ND2* | -0.70 | **0.028** |  |  |  |  |  |  |  |  |
| *MT.ND3* | -0.95 | **0.002** |  |  |  |  |  |  |  |  |
| *MT.ND4* | -0.78 | **0.006** |  |  |  |  |  |  |  |  |
| *MT.ND5* | -1.15 | **8.09E-05** |  |  |  |  |  |  |  |  |
| *MT.ND6* | -1.47 | **3.00E-06** |  |  |  |  |  |  |  |  |
| *MT.ND4L* | -0.63 | **0.015** |  |  |  |  |  |  |  |  |

Abbreviations: FC – fold change.

**Additional Table 10.** Mitochondrially encoded genes expression and their correlation with mtDNA-CN and mtDNA damage.

| **Gene** | **mtDNA-CN**  **Spearman's Rho** | **mtDNA-CN**  **P-Value** | **mtDNA damage**  **Spearman's Rho** | **mtDNA damage FDR mtDNA-CN FDR mtDNA-damage**  **P-Value** |  |  |  |  |  |
| --- | --- | --- | --- | --- | --- | --- | --- | --- | --- |
| *MT.ATP6* | -0.22 | 0.485 | 0.54 | **0.048** 0.869 0.094 |  |  |  |  |  |
| *MT.ATP8* | -0.17 | 0.604 | 0.52 | 0.062 0.869 0.094 |  |  |  |  |  |
| *MT.CO1* | -0.25 | 0.444 | 0.54 | **0.048** 0.869 0.094 |  |  |  |  |  |
| *MT.CO2* | -0.31 | 0.331 | 0.48 | 0.084 0.869 0.094 |  |  |  |  |  |
| *MT.CO3* | -0.30 | 0.342 | 0.69 | **0.008** 0.869 0.043 |  |  |  |  |  |
| *MT.CYB* | -0.06 | 0.869 | 0.48 | 0.087 0.869 0.094 |  |  |  |  |  |
| *MT.ND1* | -0.11 | 0.749 | 0.50 | 0.075 0.869 0.094 |  |  |  |  |  |
| *MT.ND2* | -0.06 | 0.852 | 0.46 | 0.097 0.869 0.097 |  |  |  |  |  |
| *MT.ND3* | -0.12 | 0.716 | 0.68 | **0.010** 0.869 0.043 |  |  |  |  |  |
| *MT.ND4* | -0.26 | 0.412 | 0.53 | 0.057 0.869 0.094 |  |  |  |  |  |
| *MT.ND5* | -0.08 | 0.817 | 0.622 | **0.020** 0.869 0.065 |  |  |  |  |  |
| *MT.ND6* | -0.17 | 0.604 | 0.71 | **0.006** 0.869 0.043 |  |  |  |  |  |
| *MT.ND4L* | -0.15 | 0.651 | 0.49 | 0.081 0.869 0.094 |  |  |  |  |  |

Abbreviations: mtDNA – mitochondrial DNA, mtDNA-CN – mitochondrial DNA copy number.

**Additional Table 11**. Nuclear-encoded genes with mitochondrial localization and correlation of their expression with mtDNA damage and mtDNA-CN.

| **Gene** | **Localization** | **mtDNA-CN**  **Spearman's Rho** | **mtDNA-CN**  **P-Value** | **mtDNA damage**  **Spearman's Rho** | **mtDNA damage FDR mtDNA-CN FDR**  **P-Value mtDNA damage** |
| --- | --- | --- | --- | --- | --- |
| *ANK2* | chromosome 4 | -0.62 | **0.037** | 0.73 | **0.005** 0.042 0.010 |
| *BCL2L1* | chromosome 20 | 0.61 | **0.040** | -0.72 | **0.005** 0.042 0.010 |
| *CLU* | chromosome 8 | -0.62 | **0.037** | 0.60 | **0.025** 0.042 0.028 |
| *CNR1* | chromosome 5 | -0.64 | **0.028** | 0.70 | **0.007** 0.042 0.011 |
| *GOT2* | chromosome 16 | 0.66 | **0.022** | -0.78 | **0.002** 0.042 0.010 |
| *POU5F1B* | chromosome 8 | 0.64 | **0.030** | -0.60 | **0.028** 0.042 0.028 |
| *MAPK10* | chromosome 4 | -0.62 | **0.035** | 0.82 | **0.001** 0.042 0.006 |
| *MAPK12* | chromosome 22 | -0.62 | **0.037** | 0.69 | **0.008** 0.042 0.012 |
| *SLIT3* | chromosome 5 | -0.66 | **0.024** | 0.76 | **0.003** 0.042 0.010 |
| *PITRM1* | chromosome 10 | 0.62 | **0.035** | -0.60 | **0.028** 0.042 0.028 |
| *UBIAD1* | chromosome 1 | 0.66 | **0.024** | -0.70 | **0.007** 0.042 0.011 |
| *VPS54* | chromosome 2 | 0.66 | **0.022** | -0.88 | **<2.2e-16** 0.042 4.18E-15 |
| *PTRH2* | chromosome 17 | 0.61 | **0.040** | 0.74 | **0.004** 0.042 0.010 |
| *EXD2* | chromosome 14 | 0.64 | **0.030** | -0.65 | **0.014** 0.042 0.018 |
| *ASAH2* | chromosome 10 | 0.61 | **0.040** | -0.72 | **0.005** 0.042 0.010 |
| *MCCC2* | chromosome 5 | 0.62 | **0.037** | -0.80 | **0.001** 0.042 0.006 |
| *SLC25A51* | chromosome 9 | 0.62 | **0.035** | -0.72 | **0.005** 0.042 0.010 |
| *MSRB3* | chromosome 12 | -0.59 | **0.046** | 0.63 | **0.019** 0.046 0.023 |
| *MTHFD2L* | chromosome 2 | 0.67 | **0.020** | -0.68 | **0.009** 0.042 0.012 |

Abbreviations: mtDNA – mitochondrial DNA, mtDNA-CN – mitochondrial DNA copy number.
